# Regulation by Nrf2 of IL-1β-induced inflammatory and oxidative response in VSMC and its relationship with TLR4

**DOI:** 10.3389/fphar.2023.1058488

**Published:** 2023-03-02

**Authors:** Zoe González-Carnicero, Raquel Hernanz, Marta Martínez-Casales, María Teresa Barrús, Ángela Martín, María Jesús Alonso

**Affiliations:** ^1^ Departamento de Ciencias Básicas de la Salud, Facultad de Ciencias de la Salud, Universidad Rey Juan Carlos, Alcorcón, Spain; ^2^ CIBER de Enfermedades Cardiovasculares, Madrid, Spain

**Keywords:** IL-1β, Nrf2, TLR4, oxidative stress, inflammation, vascular smooth muscle cells

## Abstract

**Introduction:** Vascular oxidative stress and inflammation play an important role in the pathogenesis of cardiovascular diseases (CVDs). The proinflammatory cytokine Interleukin-1β (IL-1β) participates in the vascular inflammatory and oxidative responses and influences vascular smooth muscle cells (VSMC) phenotype and function, as well as vascular remodelling in cardiovascular diseases. The Toll-like receptor 4 (TLR4) is also involved in the inflammatory response in cardiovascular diseases. A relationship between Interleukin-1β and Toll-like receptor 4 pathway has been described, although the exact mechanism of this interaction remains still unknown. Moreover, the oxidative stress sensitive transcription factor nuclear factor-erythroid 2-related factor 2 (Nrf2) promotes the transcription of several antioxidant and anti-inflammatory genes. Nuclear factor-erythroid 2-related factor 2 activators have shown to possess beneficial effects in cardiovascular diseases in which oxidative stress and inflammation are involved, such as hypertension and atherosclerosis; however, the molecular mechanisms are not fully understood. Here, we analysed the role of Toll-like receptor 4 in the oxidative and inflammatory effects of Interleukin-1β as well as whether nuclear factor-erythroid 2-related factor 2 activation contributes to vascular alterations by modulating these effects.

**Materials:** For this purpose, vascular smooth muscle cells and mice aortic segments stimulated with Interleukin-1β were used.

**Results:** Interleukin-1β induces MyD88 expression while the Toll-like receptor 4 inhibitor CLI-095 reduces the Interleukin-1β-elicited COX-2 protein expression, reactive oxygen species (ROS) production, vascular smooth muscle cells migration and endothelial dysfunction. Additionally, Interleukin-1β increases nuclear factor-erythroid 2-related factor 2 nuclear translocation and expression of its downstream proteins heme oxygenase-1, NAD(P)H:quinone oxidoreductase 1 and superoxide dismutase-2, by an oxidative stress-dependent mechanism; moreover, Interleukin-1β reduces the expression of the nuclear factor-erythroid 2-related factor 2 inhibitor Keap1. The nuclear factor-erythroid 2-related factor 2 activator tert-butylhydroquinone (tBHQ) reduces the effects of Interleukin-1β on the increased reactive oxygen species production and the expression of the proinflammatory markers (p-p38, p-JNK, p-c-Jun, COX-2), the increased cell proliferation and migration and prevents the Interleukin-1β-induced endothelial dysfunction in mice aortas. Additionally, tert-butylhydroquinone also reduces the increased MyD88 expression, NADPHoxidase activity and cell migration induced by lipopolysaccharide.

**Conclusions:** In summary, this study reveals that Toll-like receptor 4 pathway contributes to the prooxidant and proinflammatory Interleukin-1β-induced effects. Moreover, activation of nuclear factor-erythroid 2-related factor 2 prevents the deleterious effects of Interleukin-1β, likely by reducing Toll-like receptor 4-dependent pathway. Although further research is needed, the results are promising as they suggest that nuclear factor-erythroid 2-related factor 2 activators might protect against the oxidative stress and inflammation characteristic of cardiovascular diseases.

## Introduction

Cardiovascular diseases (CVDs) are one of the leading causes of morbidity and mortality worldwide (Şahin and İlgün, 2022). Vascular oxidative stress and inflammation play a significant role in the pathogenesis of CVDs, including hypertension and atherosclerosis (Senoner and Dichtl, 2019; Abbate et al., 2020; Griendling et al., 2021; Melton and Qiu, 2021). Among others, endothelial dysfunction and vascular smooth muscle cell (VSMC) alterations, such as abnormal migration and proliferation, play pivotal roles in the development of these pathologies (Wang et al., 2012; Eun et al., 2015; Badran et al., 2020; Griendling et al., 2021; Melton and Qiu, 2021).

Interleukin-1β (IL-1β) is a prototypical proinflammatory cytokine belonging to the IL-1 family (Abbate et al., 2020). IL-1β binds to its corresponding receptor and mostly signals through the myeloid differentiation factor 88 (MyD88) to facilitate downstream upregulation of inflammatory genes (Abbate et al., 2020; Melton and Qiu, 2021). Several reports suggest that IL-1β levels are increased in patients with CVDs such as hypertension and atherosclerosis, where this cytokine participates in the vascular proinflammatory and oxidative responses (Krishnan et al., 2014; Pfeiler et al., 2019; Mai and Liao, 2020; Melton and Qiu, 2021; Silveira Rossi et al., 2022). IL-1β also influences the phenotype and functions of VSMC, and induces cell migration and proliferation, participating in vascular remodelling in CVDs (Eun et al., 2015; Melton and Qiu, 2021).

On the other hand, the Toll-like receptor 4 (TLR4), a member of the family of pattern recognition receptors, participates in the inflammatory response in vascular diseases. TLR4 is expressed on the surface of several cell types, including VSMC (Nunes et al., 2019). Its activation occurs in response to both exogenous pathogen-associated molecular patterns (PAMPs) and endogenous molecules released by cells following tissue damage, called damage-associated molecular patterns (DAMPs) (Nunes et al., 2019). Although the classic TLR4 signalling pathway involves both MyD88-dependent and independent mechanisms (Liu et al., 2014), the TLR4-induced inflammatory response occurs mainly through activation of MyD88-dependent pathways (Liu et al., 2014; Biancardi et al., 2017). Signalling through TLR4 contributes to vascular inflammatory pathologies such as atherosclerosis and hypertension (De Batista et al., 2014; Yang et al., 2014; Hernanz et al., 2015). Furthermore, a relationship between IL-1β and TLR4 has recently been described, since treatment with this cytokine increases the activity of the TLR4/NF-κB pathway (Liu et al., 2014; Xu et al., 2020); however, the exact mechanism by which IL-1β interacts with this receptor is still unknown.

Reactive oxygen species (ROS) have been recognized as mediators of cellular signalling. At moderate levels, ROS are involved in physiological processes, but an excessive ROS production leads to disruption of redox signalling and to molecular damage (Xia et al., 2017; Griendling et al., 2021). To prevent ROS-induced alterations there are several antioxidant systems, among others, the nuclear factor-erythroid 2-related factor 2 (Nrf2). Nrf2 is a redox transcription factor (Yamamoto et al., 2018; Robledinos-Antón et al., 2019) which, under basal conditions, is restricted to the cytoplasm where it interacts with Kelch-like ECH-associated protein 1 (Keap1), leading to its ubiquitination (Yamamoto et al., 2018; Robledinos-Antón et al., 2019). Under stress conditions (ROS, electrophiles), Keap1 cysteines are oxidized, allowing Nrf2 to be released from its repressor Keap1 and translocate into the nucleus, wherein it heterodimerizes with small Maf proteins (sMAF) and binds to antioxidant response elements (AREs) on DNA, leading to transcription of ARE-driven genes. This set of genes regulates the expression of phase II detoxifying enzymes, including NAD(P)H:quinone oxidoreductase 1 (NQO1) or glutathione peroxidase (GPx), and antioxidant proteins such as heme oxygenase-1 (HO-1) (Yamamoto et al., 2018; Robledinos-Antón et al., 2019). Nrf2 has been related to different CVDs such as hypertension and atherosclerosis (Alonso-Piñeiro et al., 2021; Tanase et al., 2022). Thus, Nrf2 downregulation in stroke-prone spontaneously hypertensive rats (SHRSP) contributes to the increased oxidative stress and vascular dysfunction observed in this model (Lopes et al., 2015); however, patients who suffer from atherosclerosis have higher levels of Nrf2/HO-1 than healthy subjects (Fiorelli et al., 2019), suggesting that Nrf2 activation is an adaptive mechanism against oxidative stress characteristic of this CVDs.

Tert-butylhydroquinone (tBHQ) is a Nrf2 activator with antioxidant properties that enhances Nrf2-mediated transcription by promoting dissociation of Nrf2-Keap1 (Zhang and Hannink, 2003). Activators of Nrf2 have shown to have a beneficial effect in pathologies involving oxidative stress and inflammation (Xia et al., 2017; Robledinos-Antón et al., 2019). Thus, sulforaphane reduces ROS production both in vessels and VSMC from SHRSP (Lopes et al., 2015), and resveratrol, which also activates Nrf2, reduces ROS generation and lipid peroxidation, thus contributing to prevent from atherosclerosis (Parsamanesh et al., 2021). On the other hand, it has been described that tBHQ prevents microvascular endothelial dysfunction and remodelling and reduces blood pressure in angiotensin II-induced hypertension (Wang et al., 2018). Based on the mentioned data, Nrf2 signalling pathway is currently considered an important defence mechanism against several CVDs; however, the mechanisms underlying the preventive effects of Nrf2 are barely known.

The aim of this study was to analyse whether TLR4 pathway contributes to the proinflammatory and prooxidant IL-1β-induced effects; in addition, the result of Nrf2 activation by tBHQ on these IL-1β-mediated effects and the possible mechanisms involved were also evaluated.

## Materials and methods

### Ethic statements

All experimental procedures were approved by the Ethical Committee of Research of the Universidad Autónoma de Madrid and Dirección General de Medio Ambiente, Comunidad de Madrid, Spain (PROEX 183.2/20). Animal care and experimental procedures conformed to the current Spanish laws (RD 53/2013) and are also conformed to the Directive 2010/63/EU of the European Parliament for animal experiments. The studies also comply with the ARRIVE guidelines for reporting experiments involving animals. C57BL/6 mice were obtained from colonies maintained at the Animal Quarters of the Facultad de Medicina of the Universidad Autónoma de Madrid. During treatment, mice were housed with constant room temperature, humidity and light cycle (12-h light/dark) and they had free access to tap water and were fed with standard mice chow *ad libitum*.

### Cell culture

Experiments were performed using the mouse aortic VSMC line MOVAS (ATCC^®^ CRL-2797™, Manassas, VA, United States). Cells were maintained in High Glucose Dulbecco’s Modified Eagle Medium [High Glucose-DMEM (Sigma Chemical Co., St. Louis, MO, United States)] supplemented with 10% (v/v) fetal bovine serum (FBS) and 0.2 mg/mL of the G-418 solution (Roche Diagnostics, Manheim, Germany). VSMC were incubated at 37°C in a humidified 5% CO_2_ atmosphere. Cells from passages 3-12 were used for the experiments. Cells were starved in High Glucose-DMEM, 0% FBS and 0.2 mg/mL of the G-418 solution. Afterwards, cells were stimulated with 10 ng/mL IL-1β for 1 h with or without pretreatment for 24 h with the Nrf2 activator tBHQ (20 μM) or for 1 h with apocynin (30 μM), catalase (1,000 U/mL) or the general intracellular TLR4 inhibitor CLI-095 (1 μM). In other set of experiments, cells were stimulated with hydrogen peroxide (H_2_O_2_, 100 μM, 1 h) or lipopolysaccharide (LPS, 10 μg/mL, 3 h) in the absence or the presence of tBHQ (20 μM, 24 h).

### Western blot for protein expression analysis

Protein expression was determined by Western blot in whole-cell lysates (15–30 µg) or nuclear and cytosolic fractions (15–20 µg) from VSMC. Nuclear and cytosolic extracts were obtained as previously described (Martín et al., 2012). Proteins were separated by 7.5% or 12% SDS-PAGE and transferred to polyvinyl difluoride membranes that were incubated with antibodies for Nrf2, HO-1, NQO1, SOD1, SOD2, Keap1, TLR4, MyD88, COX-2, p-JNK, p-p38 and p-c-Jun (Table 1). After washing, membranes were then incubated with secondary anti-rabbit (1:2,000; Bio-Rad Laboratories, Hercules, CA, United States) or anti-mouse antibodies (1:4,000; Bio-Rad Laboratories) conjugated to horseradish peroxidase. Proteins were detected using a horseradish peroxidase-luminol/enhancer chemiluminescence system (Bio-Rad Laboratories). The same membrane was used to determine the expression of α-tubulin, p38 and JNK in total extracts or GAPDH and TBP in cytosolic and nuclear extracts, respectively, as loading control by using mouse monoclonal or rabbit polyclonal antibodies (Table 1). Immunoblot signals were quantified using the Image Lab Software version 6.0 (Bio-Rad Laboratories). For protein expression, the ratio between signals on the immunoblot corresponding to the protein studied and that of tubulin, p-38, JNK or TBP was calculated. The protein expression in control cells was assigned the value of 1 to compare with treated cells.

**TABLE 1:** Summary of antibodies.

Antibody	Dilution	Source	Selling company	Catalog number
Nrf2	1:1,000	Rabbit monoclonal	Cell Signaling Technology (Danvers, MA, United States)	12721
HO-1	1:1,000	Rabbit polyclonal	ENZO Life Sciences (Lausen, Switzerland)	ADI-SPA-895
NQO1	1:500	Mouse monoclonal	Santa Cruz Biotechnology (Santa Cruz, CA, United States)	sc-32793
SOD1	0.05 μg/mL	Rabbit polyclonal	ENZO Life Sciences	ADI-SOD-101
SOD2	0.005 μg/mL	Rabbit polyclonal	ENZO Life Sciences	ADI-SOD-111
Keap1	1:1,000	Rabbit monoclonal	Cell Signaling Technology	8047
TLR4	1:500	Mouse monoclonal	Santa Cruz Biotechnology	sc-293072
MyD88	1:1,000	Rabbit polyclonal	Abcam (Cambridge, United Kingdom)	ab2064
COX-2	1:150	Rabbit polyclonal	Cayman Chemical (Ann Arbor, MI, United States)	160126
p-JNK	1:500	Rabbit polyclonal	Cell Signaling Technology	9251
JNK	1:1,000	Rabbit monoclonal	Cell Signaling Technology	9258
p-p38	1:1,000	Mouse monoclonal	Cell Signaling Technology	9216
p38	1:2,000	Mouse monoclonal	Cell Signaling Technology	9217
p-c-Jun	1:500	Rabbit polyclonal	Santa Cruz Biotechnology	sc-16312
Tubulin	1:20,000	Mouse monoclonal	Sigma Chemical Co.	T5168
TBP	1:1,000	Rabbit polyclonal	Santa Cruz Biotechnology	sc-204
GAPDH	1:1,000	Mouse monoclonal	Calbiochem (Temecula, CA, United States)	CB1001

### Detection of ROS production: Fluorescence microscopy—Flow cytometry

The oxidative fluorescent dye dihydroethidium (DHE) and the colorant 2′,7′-dichlorofluorescein diacetate (DCFH-DA) were used to evaluate superoxide anion (O_2_^.^^−^) and H_2_O_2_ production, respectively. Hydroethidine is able of freely cross cell membranes and oxidize in the presence of O_2_^.^^−^ to ethidium bromide, which is trapped inside the cell due to its ability to intercalate into DNA. Ethidium bromide is excited at a wavelength of 546 nm and has an emission spectrum at 600–700 nm. DCFH-DA is a permeable dye able of diffusing through the cell membrane. DCFH-DA is degraded by intracellular esterases to 2′-7′ dichlorofluorescein (DCF), which binds to intracellular H_2_O_2_ and emits fluorescence at a wavelength of 535 nm when excited at a wavelength of 485 nm.

#### Fluorescence microscopy

Briefly, VSMC were plated onto glass coverslips placed in 12-well plates and cultured as described above. Cells at a confluence of 40%–60% were stimulated with 10 ng/mL IL-1β or 100 μM H_2_O_2_ for 1 h in the absence or the presence of 20 μM tBHQ for 24 h. After that, cells were then incubated with 10 μM DHE in cell culture medium for 30 min at 37°C. The images were then acquired using a fluorescence microscope (Zeiss Axioplan 2, Carl Zeiss Microscopy, LLC, Thornwood, NY, United States) and a cool fluorescence colour camera (Leica DFC7000T). The fluorescence intensity values of 10–12 nuclei per experiment were measured using ImageJ software (http://rsb.info.nih.gov/ij). Data were expressed as an increase in the fluorescence intensity related to the control value.

#### Flow cytometry

Briefly, VSMC were plated in 12-well plates, cultured as described above, and stimulated with 10 ng/mL IL-1β or 100 μM H_2_O_2_ for 1 h in the absence or the presence of 20 μM tBHQ for 24 h or 1 μM CLI-095 for 1 h. Cells were then incubated with 10 μM DHE or 20 μM DCFH-DA in cell culture medium for 30 min at 37°C. Next, cells were trypsinized and collected in tubes for centrifugation. After two washes with PBS, cells in the pellet were resuspended in 500 μl PBS and analysed by flow cytometry using a Beckman Coulter Cytomics FC500 MPL cytometer (Beckman Coulter, Miami, FL, United States). Data were expressed as an increase in the fluorescence intensity related to the control value.

### Lucigenin assay

The superoxide anion generated by nicotinamide adenine dinucleotide phosphate (NADPH) oxidase activity was determined using a chemiluminescence assay using lucigenin and NADPH. For this, VSMC were treated with 10 ng/mL IL-1β or 100 μM H_2_O_2_ for 1 h, or 10 μg/mL LPS for 3 h, with or without pretreatment with tBHQ 20 μM for 24 h, and then they were homogenized in a lysis buffer (50 mM KH_2_PO_4_, 1 mM ethylene glycol tetra acetic acid—EGTA, 150 mM sucrose, pH = 7.4). The resulting homogenate was then transferred to a 96 wells plate together with the lysis buffer and lucigenin (5 µM). The assay was performed by duplicate. Basal luminescence was measured every 1.8 s during 3 min in a plate luminometer (GloMax®-Multi Detection System: Promega, Madison, WI, United States). The reaction was initiated by adding NADPH (100 µM) to the samples in a final volume of 300 μl. Luminescence was determined every 1.8 s for 3 min. Baseline activity in the absence of NADPH was subtracted. Activity was expressed as relative light units per μg of protein. Protein concentration was determined by using the Micro BCA™ protein assay kit (Thermo Fisher Scientific, Rockford, IL, United States). Data were expressed as an increase in the luminescence related to the control cells.

### *In vitro* wound healing assay

To evaluate the effect of tBHQ and CLI-095 on IL-1β- and LPS-induced migration, wound healing assay was used. For this, cells were seeded and cultured in 12-well plates. Once the cells reached a confluence of 90%, the medium was removed, a wound was made with a P10 pipette tip, and a line was drawn through the centre of the wells, perpendicular to the wound. After two washes with PBS (to wash away any cell debris remaining in the wound area), serum-free medium was added. A picture was taken at time zero at the site of intersection of the line and the wound. Then, VSMC were stimulated with 10 ng/mL IL-1β or 100 μM H_2_O_2_ for 24 h in the absence or presence of 20 μM tBHQ or 1 μM CLI-095. After 24 h, we took a picture in the same location. ImageJ software was used to determine the area of wound closure compared to time 0 for the stimulus and with respect to the control situation.

### Cell proliferation assay

3-(4,5-dimethyl-2-thiazolyl)-2,5-diphenyltetrazolium bromide (MTT) was used to evaluate VSMC proliferation. For this, cells were seeded in 12-well plates. Once the cells reached a confluence of 50%–60%, VSMC were starved in serum-free medium and stimulated with 10 ng/mL IL-1β or 100 μM H_2_O_2_ for 24 h in the absence or presence of 20 μM tBHQ or 1 μM CLI-095. Then, the medium was removed and MTT solution was added for 3 h. MTT is transformed into formazan crystals (insoluble in water) by the action of mitochondrial dehydrogenases in living cells. The blue formazan crystals are solubilized with DMSO, resulting in a colorimetric reaction leading to the appearance of a purple coloration. The absorbance was measured at 570 nm using a FLUOstar omega spectrophotometer (BMG Labtech, Germany).

### Vascular function

Some experiments were performed in aortic segments from 3-month C57BL/6 male mice. Thoracic aorta was divided into four segments 2 mm in length, each one incubated for 24 h without or with 10 ng/mL IL-1β, 20 μM tBHQ and IL-1β+tBHQ or IL-1β, 1 μM CLI-095 and IL-1β+CLI-095 in incubation medium (DMEM low glucose supplemented with 1% (v/v) FBS, 1% Penicillin-Streptomycin and 1% Glutamine). Afterwards, segments were transferred to a wire myograph to measure vascular reactivity. After a 30-min equilibration period in oxygenated Krebs-Henseleit solution (KHS, in mM: 115 NaCl, 25 NaHCO_3_, 4.7 KCl, 1.2 MgSO_4_.7H_2_O, 2.5 CaCl_2_, 1.2 KH_2_PO_4_, 11.1 glucose, and 0.01 Na_2_EDTA) bubbled with a 95% O_2_-5% CO_2_ mixture (pH = 7.4), arterial segments were stretched to their optimal lumen diameter for active tension development. Segment´s contractility was tested by an initial exposure to a high K^+^ solution (120 mM K^+^-KHS, which was identical to KHS except that NaCl was replaced by KCl on an equimolar basis). Then, endothelium-dependent vasodilation was analysed by performing a single cumulative concentration-response curve to ACh (1 nM—10 μM) in arteries precontracted with phenylephrine (Phe) at a concentration that produce approximately 50% of the contraction induced by K^+^-KHS.

### Statistical analysis

All values are expressed as mean ± standard error (S.E.M.); *n* denotes the number of animals or the number of different cultures (each one obtained from one different passage) used in each experiment. For cell culture experiments, data are expressed as n-fold increase relative to the average value of controls in each plate or each blot. For vascular reactivity experiments, vasodilator responses induced by ACh were expressed as the % of the previous tone in each case.

Results were analysed by using paired Student’s *t*-test, one-way or two-way ANOVA followed by Bonferroni post-test by using GraphPad Prism Software (San Diego, CA, United States). Values were considered to be significant when *p*-value is less than 0.05.

### Drugs/chemicals and antibodies

FBS, Penicillin-Streptomycin, Glutamine, IL-1β, tBHQ, LPS, H_2_O_2,_ apocynin, catalase, DHE, DCFH-DA, MTT, Phe, ACh, Lucigenin and NADPH were obtained from Sigma Chemical Co. CLI-095 was obtained from InvivoGen (San Diego, CA, United States). All drugs were dissolved in distilled water except for tBHQ, CLI-095, DHE, DCFH-DA, which were dissolved in DMSO, and Lucigenin, which was dissolved in acetic acid. Neither DMSO nor acetic acid have any effect on VSMC.

## Results

### TLR4 pathway is involved in Interleukin-1β effects

As mentioned, IL-1β is associated to increased ROS production; the primary enzymatic sources of cardiovascular ROS are NADPH oxidases. Accordingly, IL-1β (10 ng/mL, 1 h) increased the NADPH oxidase activity (Supplementary Figure S1A) and the subsequent production of hydrogen peroxide and superoxide anion in VSMC (Supplementary Figures S1B–D). In addition, treatment of VSMC with IL-1β induced the nuclear translocation of the AP-1 subunit phospho-c-Jun and the COX-2 protein expression (Supplementary Figure S2). In this study we also prove that oxidative stress is involved in the proinflammatory effects mediated by this cytokine. Thus, IL-1β-elicited nuclear translocation of p-c-Jun was reduced by the antioxidant apocynin (30 µM) and the H_2_O_2_ scavenger catalase (1,000 U/mL) (Supplementary Figures S2A, B). Similarly, both drugs reduced the IL-1β-induced COX-2 expression (Supplementary Figures S2C, D). Neither apocynin nor catalase modified the nuclear p-p-Jun or COX-2 expressions (Supplementary Figure S2). These results confirm that ROS participate in IL-1β-induced activation of AP-1 and COX-2 in VSMC.

**FIGURE 1:**
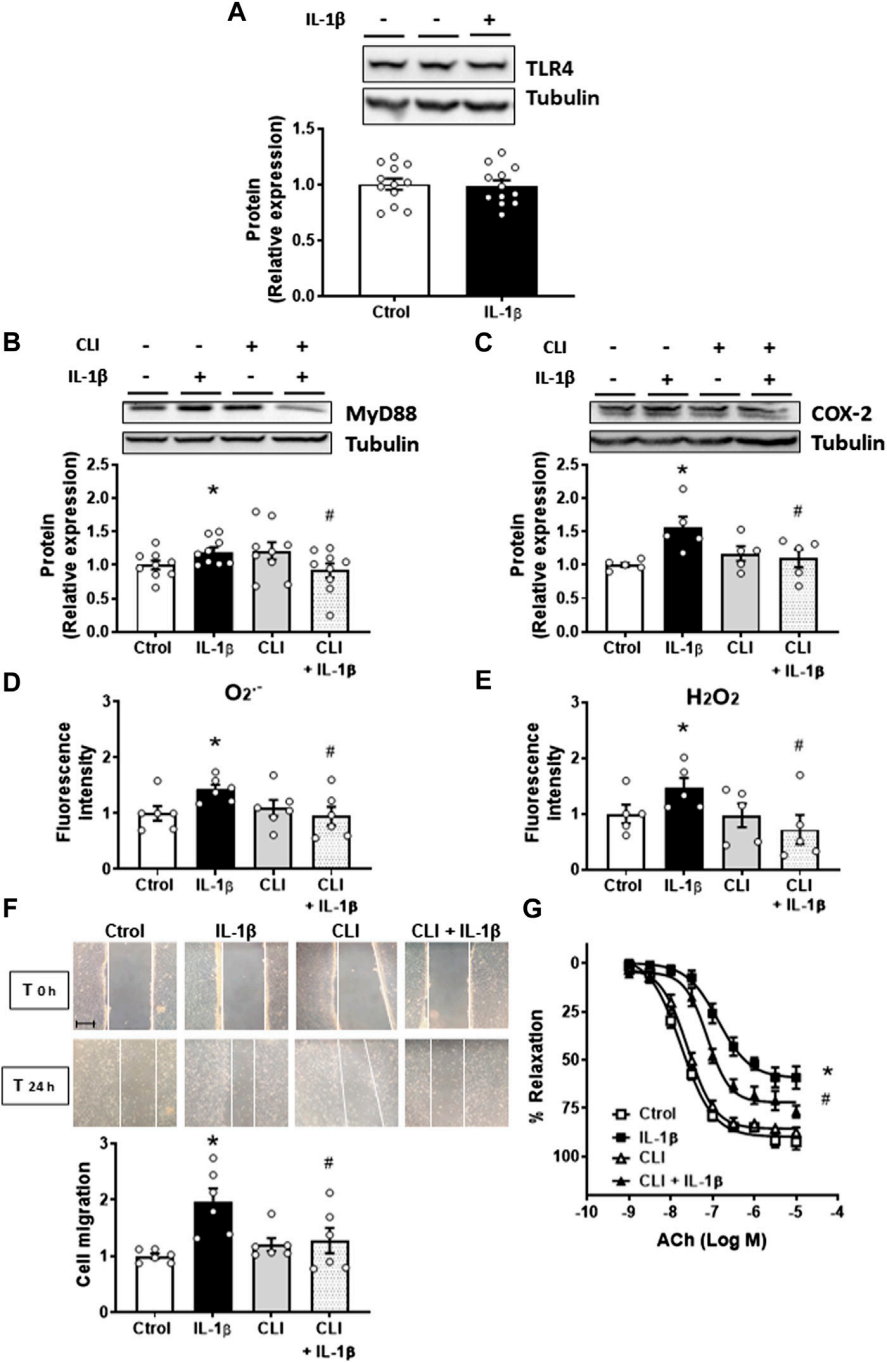
Role of TLR4 pathway on the IL-1β-induced effects. Effect of Interleukin-1β (IL-1β, 10 ng/mL) on TLR4 expression **(A)**. Effect of CLI-095 (1 µM), IL-1β and CLI-095 + IL-1β on myeloid differentiation factor 88 (MyD88) and cyclooxygenase-2 (COX-2) protein expression **(B,C)**. Effect of CLI-095 on IL-1β-induced superoxide anion (O_2_^.-^) and hydrogen peroxide (H_2_O_2_) production evaluated by flow cytometry **(D,E)** and cell migration **(F)** in vascular smooth muscle cells. Representative blots are shown in upper panels. Images of cell migration by wound healing assay are included; bar scale represents 50 μm. *p < 0.05 vs. control; #*p* < 0.05 vs. IL-1β by Student’s *t*-test (*n* = 5–12). Effect of CLI-095, IL-1β and CLI-095 + IL-1β on acetylcholine (ACh)-induced relaxation in mice aortic segments precontracted with phenylephrine **(G)**. *p < 0.05 vs. control; #*p* < 0.05 vs. IL-1β by two-way ANOVA followed by Bonferroni’s post test (*n* = 5). See methods for the incubation times.

Next, we analysed the effect of IL-1β on the TLR4 signalling pathway. Treatment of mouse aortic VSMC with IL-1β did not modify TLR4 expression (Figure 1A); however, it increased the expression of its adapter protein MyD88 (Figure 1B). The effect of IL-1β on MyD88 protein expression was prevented by using the general intracellular TLR4 inhibitor CLI-095 (1 μM) (Figure 1B), suggesting that the TLR4/MyD88-dependent signalling pathway was activated, at least in part, by IL-1β in VSMC. Next, we analysed the contribution of TLR4 to the observed effects of IL-1β. The inhibition of TLR4 by CLI-095 reduced the increase in COX-2 expression induced by IL-1β (Figure 1C). Besides, CLI-095 also reduced the IL-1β-increased O_2_^.-^ and H_2_O_2_ production (Figures 1D, E). CLI-095 alone did affect neither COX-2 and MyD88 expressions nor O_2_^−^ and H_2_O_2_ production (Figures 1B, C). Moreover, we found that IL-1β (10 ng/mL, 24 h) increased VSMC migration, and this effect was reduced by blocking the TLR4 pathway; CLI-095 did not affect cell migration (Figure 1F). The IL-1β-induced VSMC proliferation was also prevented by CLI-095 (results not shown).

Thereafter, whether TLR4 inhibition interferes with the IL-1β effect on vasodilator responses were also analysed. Incubation of mouse aortas with IL-1β (10 ng/mL, 24 h) induced endothelial dysfunction, while cotreatment with the TLR4 antagonist improved ACh-induced endothelium-dependent vasodilatation (Figure 1G); however, IL-1β did not affect the response to K-KHS, regardless of the presence or absence of CLI-095 (data not shown). Furthermore, CLI-095 alone did not modify ACh-induced relaxation (Figure 1G). Taken together, all these results indicate that the TLR4 pathway contributes to the IL-1β-mediated effects.

### Interleukin-1β-induced Nrf2 activation is dependent on reactive oxygen species production

**FIGURE 2:**
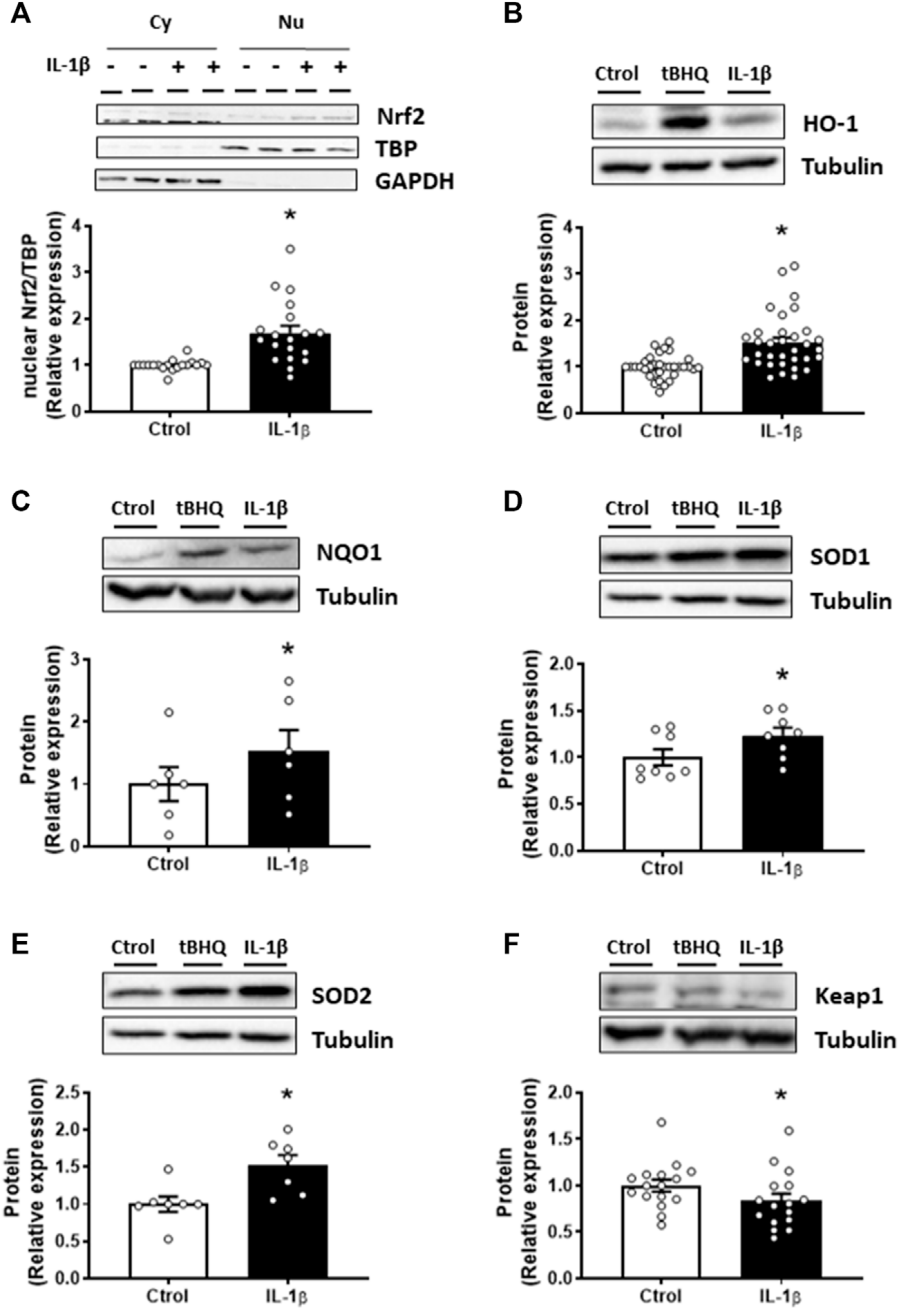
Effect of IL-1β on the Nrf2 pathway. Effect of Interleukin-1β (IL-1β, 10 ng/mL, 1 h) on the nuclear Nrf2 (nuclear factor-erythroid 2-related factor 2) protein expression **(A)** in vascular smooth muscle cells; a representative blot of the cytosolic (Cy) and nuclear (Nu) expression is also shown; nuclear TATA-binding protein (TBP) and cytosolic GAPDH expressions are also shown to guarantee the successful cellular fractioning. Effect of IL-1β on heme oxygenase-1 [HO-1, **(B)**], NAD(P)H:quinone oxidoreductase 1 [NQO1, **(C)**], superoxide dismutase 1 [SOD1, **(D)**], superoxide dismutase 2 [SOD2, **(E)**] and Keap1 **(F)** protein expression. Representative blots are shown in upper panels; the same loading control for SOD1 and SOD2 protein expression was used. Tert-butylhydroquinone (tBHQ, 20 μM, 24 h) was used as positive control. *p < 0.05 vs. control by Student’s *t*-test (*n* = 6–30).

Later, we analysed the effect of IL-1β on the Nrf2 pathway. Treatment of VSMC with IL-1β (10 ng/mL, 1 h) increased the Nrf2 nuclear translocation (Figure 2A); in addition, IL-1β also enhanced the expression of its downstream proteins, HO-1, NQO1, SOD1, and SOD2 (Figures 2B–E). Importantly, IL-1β reduced the expression of the Nrf2 inhibitory protein Keap1 (Figure 2F).

**FIGURE 3:**
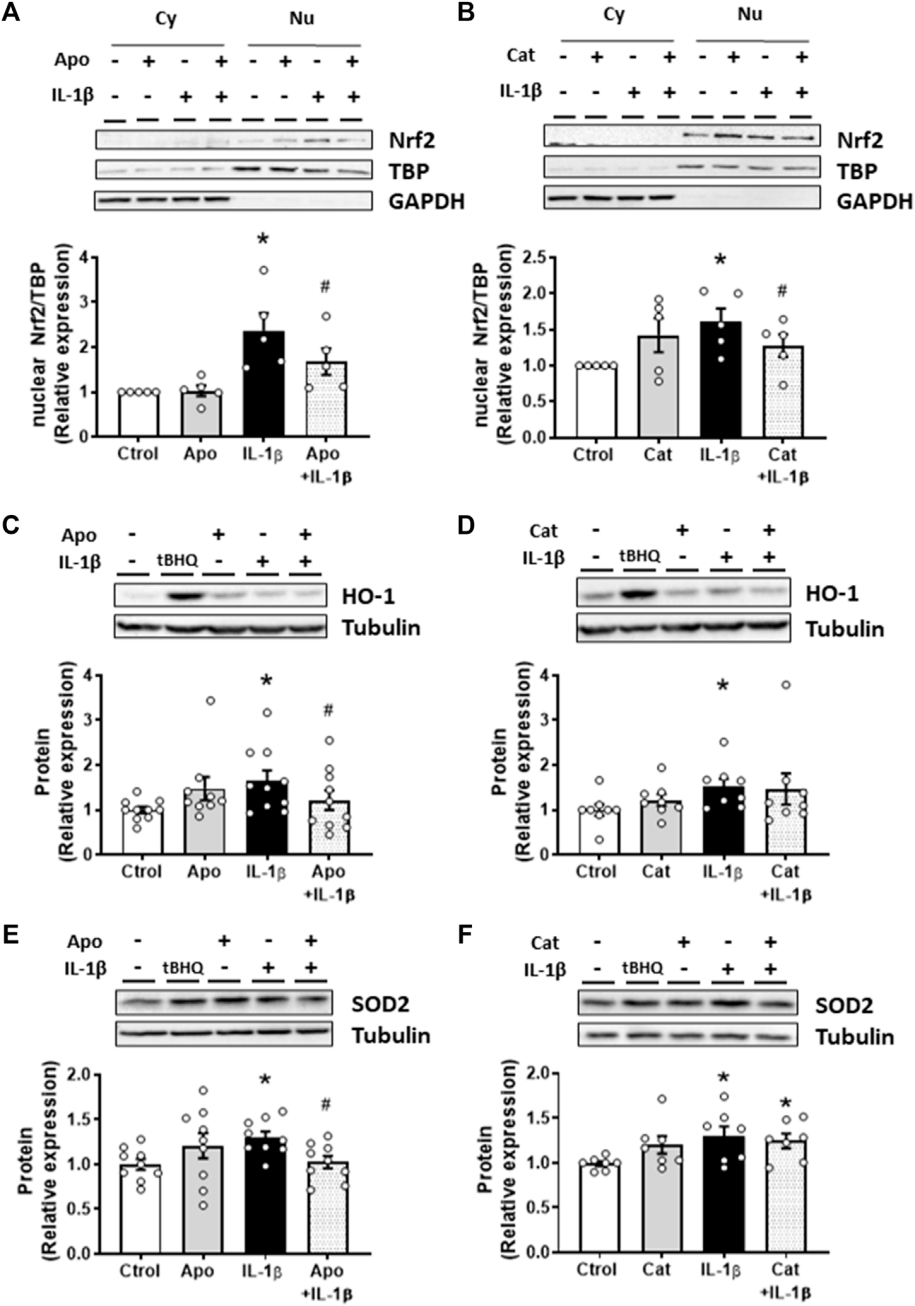
Role of oxidative stress in the IL-1β-induced effects on the Nrf2 pathway. Effect of apocynin (Apo, 30 μM, 2 h) and catalase (Cat, 1,000 U/mL, 2 h) on the nuclear Nrf2 (nuclear factor-erythroid 2-related factor 2) protein expression **(A,B)** induced by Interleukin-1β (IL-1β, 10 ng/mL, 1 h) in vascular smooth muscle cells; a representative blot of the cytosolic (Cy) and nuclear (Nu) expression is also shown; nuclear TATA-binding protein (TBP) and cytosolic GAPDH expressions are also shown to guarantee the successful cellular fractioning. Effect of Apo and Cat on heme oxygenase-1 [HO-1, **(C,D)**] and superoxide dismutase 2 [SOD2, **(E,F)**] protein expressions induced by IL-1β. Representative blots are shown in upper panels. Tert-butylhydroquinone (tBHQ, 20 μM, 24 h) was used as positive control. *p < 0.05 vs. control; #*p* < 0.05 vs. IL-1β by Student’s *t*-test (*n* = 6–10).

As described above, IL-1β increased the NADPH oxidase activity and the subsequent ROS production in VSMC. Therefore, the involvement of oxidative stress in the effects of IL-1β on Nrf2 pathway was studied. IL-1β-induced Nrf2 nuclear translocation was reduced by apocynin (30 µM) and catalase (1,000 U/mL) (Figures 3A, B); similarly, apocynin reduced the effect of IL-1β on HO-1 and SOD2 protein expression (Figures 3C, E). However, catalase did reduce the IL-1β-induced expression neither of HO-1 nor of SOD2 (Figures 3D, F). Neither apocynin nor catalase modified the proteins studied (Figure 3). In addition, treatment of VSMC with H_2_O_2_ (100 μM, 1 h) increased the Nrf2 nuclear translocation (Supplementary Figure S3A) as well as the protein expression of HO-1 and NQO1 (Supplementary Figures S3B, C). All together, these results show that ROS participate in Nrf2 activation by IL-1β.

### Nrf2 activation downregulates Interleukin-1β prooxidant and proinflammatory effects

**FIGURE 4:**
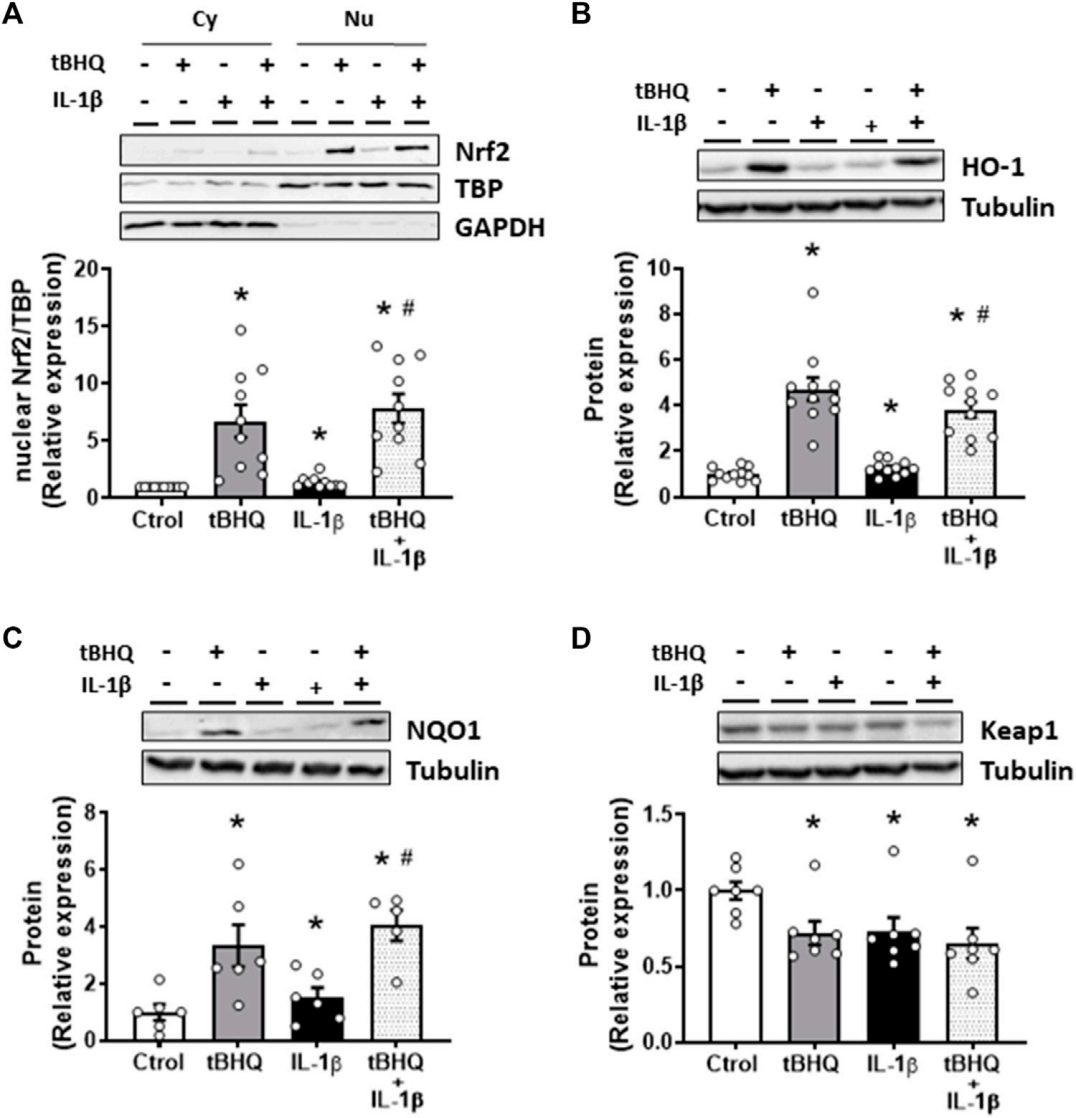
Modulation by Nrf2 activation of the IL-1β-induced effects on the Nrf2 pathway. Effect of tert-butylhydroquinone (tBHQ, 20 μM, 24 h), Interleukin-1β (IL-1β, 10 ng/mL, 1 h) and tBHQ + IL-1β on the nuclear Nrf2 (nuclear factor-erythroid 2-related factor 2) protein expression **(A)** in vascular smooth muscle cells; a representative blot of the cytosolic (Cy) and nuclear (Nu) expression is also shown; nuclear TATA-binding protein (TBP) and cytosolic GAPDH expressions are also shown to guarantee the successful cellular fractioning. Effect of tBHQ, IL-1β and tBHQ + IL-1β on heme oxygenase-1 [HO-1, **(B)**], NAD(P)H:quinone oxidoreductase 1 [NQO1, **(C)**] and Keap1 **(D)** protein expressions. Representative blots are shown in upper panels. *p < 0.05 vs. control; #*p* < 0.05 vs. IL-1β by Student’s *t*-test (*n* = 6–10).

We next analysed the effect of Nrf2 activation on IL-1β-induced effects. For this, the Nrf2 activator tBHQ was used. As expected, tBHQ (20 µM) increased the nuclear translocation of Nrf2 as well as the protein levels of HO-1 and NQO1, while it reduced the expression of Keap1 (Figure 4). Furthermore, pretreatment of VSMC for 24 h with the Nrf2 activator tBHQ increased the IL-1β-induced Nrf2 nuclear translocation as well as the HO-1 and NQO1 protein expression, although the levels reached were similar to those of tBHQ alone (Figures 4A–C). Additionally, tBHQ plus IL-1β reduced Keap1 protein expression, being this effect similar to that induced by either IL-1β or tBHQ alone (Figure 4D).

**FIGURE 5:**
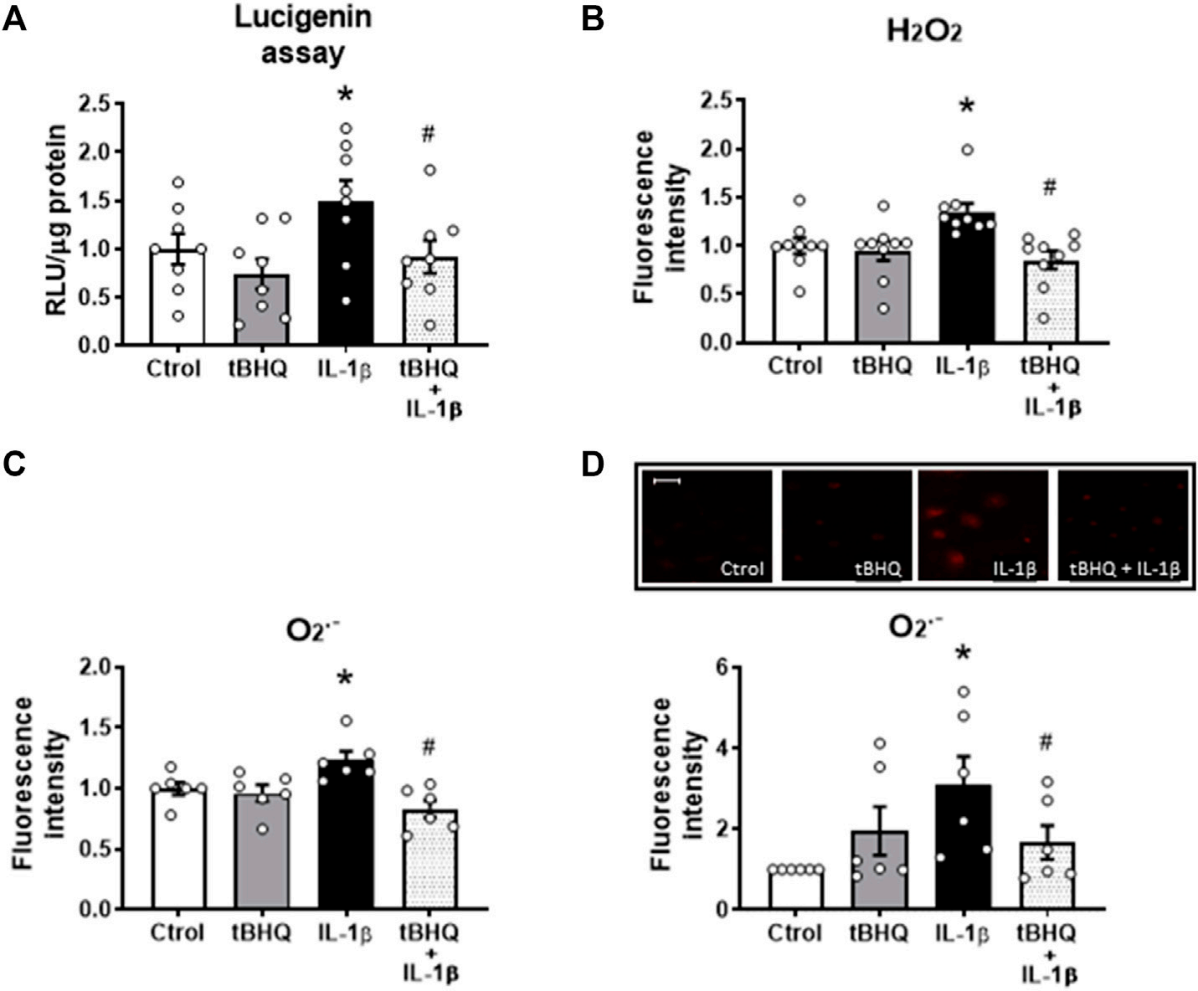
Modulation by Nrf2 activation of the IL-1β-induced effects on oxidative stress. Effect of tert-butylhydroquinone (tBHQ, 20 μM, 24 h), Interleukin-1β (IL-1β, 10 ng/mL, 1 h) and tBHQ + IL-1β on NAPDH oxidase activity **(A)**, hydrogen peroxide [H_2_O_2_, **(B)**] and superoxide anion (O_2_^.-^) production evaluated by flow cytometry **(C)** and by fluorescence microscopy **(D)** in vascular smooth muscle cells; representative fluorescent photomicrographs are also shown; images were captured with a fluorescence microscope; bar scale represents 100 μm. *p < 0.05 vs. control; #*p* < 0.05 vs. IL-1β by Student’s *t*-test (*n* = 6–10).

Next, we investigated the effect of Nrf2 activation on the IL-1β-elicited prooxidant effects. tBHQ did modify neither the basal NADPH oxidase activity nor the production of both hydrogen peroxide and superoxide anion (Figure 5); however, it reduced the increased IL-1β-induced NADPH oxidase activity (Figure 5A) as well as the IL-1β-elicited increase of both H_2_O_2_ and O_2_^.^^−^ production (Figures 5B–D). In addition, we evaluated whether Nrf2 activation affects the H_2_O_2_-elicited effects. Pretreatment of VSMC for 24 h with 20 µM tBHQ increased the H_2_O_2_-induced Nrf2 nuclear translocation (Supplementary Figure S4A) and the HO-1 and NQO1 protein expression (Supplementary Figures S4B, C), although these effects were similar to those caused by tBHQ alone. Furthermore, tBHQ reduced the H_2_O_2_-elicited NADPH oxidase activity and O_2_^.^^−^ production (Supplementary Figures S4D–F).

**FIGURE 6:**
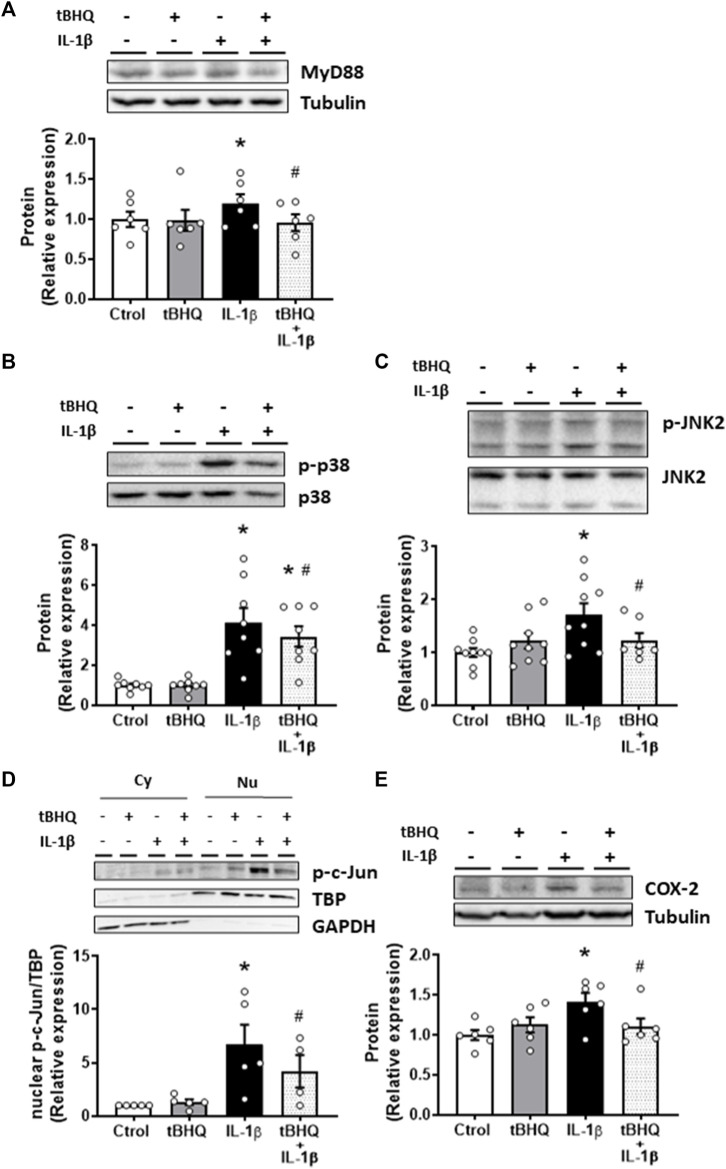
Modulation by Nrf2 activation of IL-1β-induced-effects on proinflammatory markers. Effect of tert-butylhydroquinone (tBHQ, 20 μM, 24 h), Interleukin-1β (IL-1β, 10 ng/mL, 1 h) and tBHQ + IL-1β on myeloid differentiation factor 88 [MyD88, **(A)**], p-p38 **(B)**, p-JNK2 **(C)**, nuclear p-c-Jun **(D)** and cyclooxygenase-2 [COX-2, **(E)**] protein expression in vascular smooth muscle cells. Representative blots are shown in upper panels. A representative blot of the cytosolic (Cy) and nuclear (Nu) expression of p-c-Jun is also shown **(D)**; nuclear TATA-binding protein (TBP) and cytosolic GAPDH expressions are also shown to guarantee the successful cellular fractioning *p < 0.05 vs. control; #*p* < 0.05 vs. IL-1β by Student’s *t*-test (*n* = 5–12).

Thereafter, we explored the effect of Nrf2 activation on IL-1β-elicited proinflammatory effects. Pretreatment of VSMC for 24 h with the Nrf2 activator tBHQ did not modify the protein expression of MyD88, p-p38, p-JNK2, nuclear p-c-Jun or COX-2, but it reduced the IL-1β-induced increase of these proteins, as shown in Figure 6. On the other hand, we found that bacterial LPS, the main TLR4 ligand, increased MyD88 protein expression, and this increase was reduced by tBHQ (Supplementary Figure S5A); in addition, tBHQ reduced the LPS-elicited increase of NADPH oxidase activity and cell migration (Supplementary Figures S5B, C). Our results, therefore, suggest that Nrf2 activation protects against the detrimental prooxidant and proinflammatory effects of IL-1β, probably through TLR4 pathway modulation.

### Nrf2 activation protects against Interleukin-1β induced VSMC migration and proliferation, and endothelial dysfunction

**FIGURE 7:**
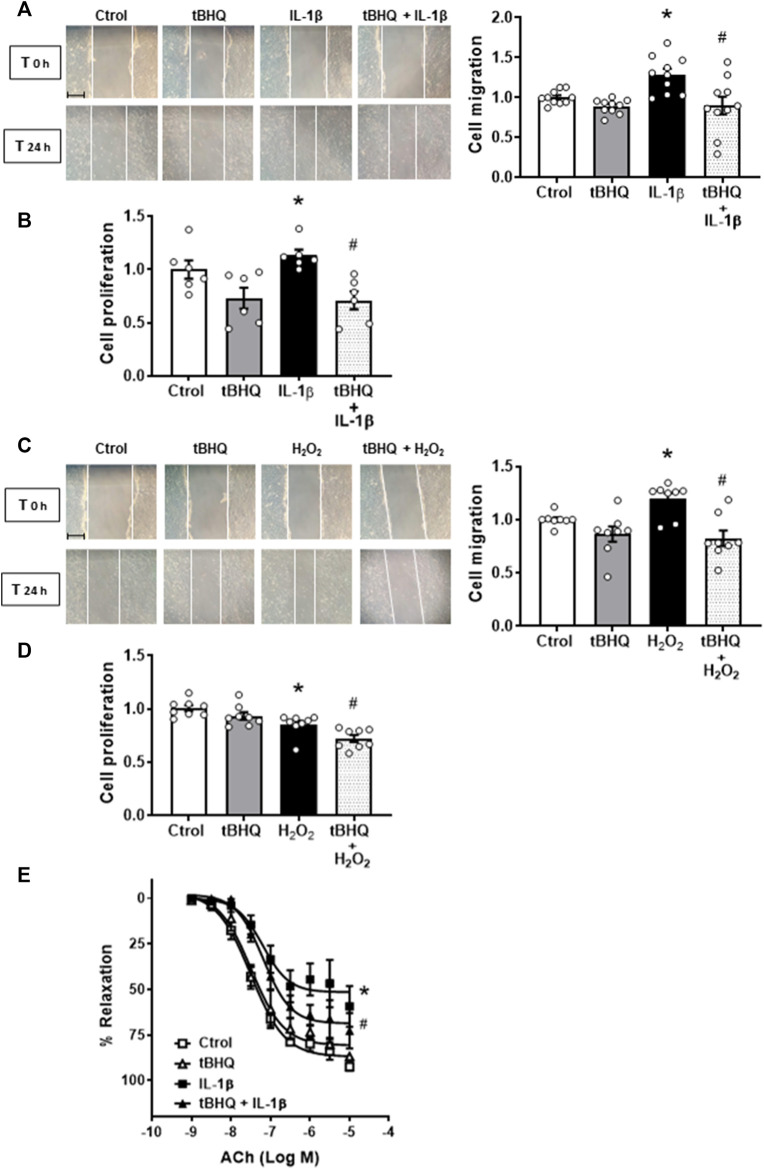
Modulation by Nrf2 activation of the IL-1β- and H_2_O_2_-induced effects on cell migration and proliferation as well as on IL-1β-induced endothelial dysfunction. Effect of tert-butylhydroquinone (tBHQ, 20 μM, 24 h), Interleukin-1β (IL-1β, 10 ng/mL, 1 h) and tBHQ + IL-1β **(A,B)** and tBHQ, hydrogen peroxide (H_2_O_2_, 100 μM, 1 h) and tBHQ + H_2_O_2_ **(C,D)** on cell migration and proliferation in vascular smooth muscle cells. Images of cell migration by wound healing assay are included; bar scale represents 50 μm. *p < 0.05 vs. control; #*p* < 0.05 vs. IL-1β by Student’s *t*-test (*n* = 6–10). Effect of tBHQ, IL-1β (10 ng/mL, 24 h) and tBHQ + IL-1β on acetylcholine (ACh)-induced relaxation in mice aortic segments precontracted with phenylephrine **(E)**. *p < 0.05 vs. control; #*p* < 0.05 vs. IL-1β by two-way ANOVA followed by Bonferroni’s post test (*n* = 4).

The activator of Nrf2 alone did modify neither cell migration nor proliferation; however, it reduced the VSMC migration and proliferation induced by incubation for 24 h with 10 ng/mL IL-1β (Figures 7A, B). In addition, tBHQ (20 μM) also reduced the cell migration elicited by H_2_O_2_ (100 μM, 24 h), as shown in Figure 7C. In contrast, H_2_O_2_ reduced cell proliferation, which was further decreased by the Nrf2 activator tBHQ (Figure 7D).

Finally, the effect of Nrf2 activation on IL-1β-induced endothelial dysfunction was also analysed. As shown in Figure 7E, tBHQ did not modify the vasodilator response induced by ACh, but it partially prevented the IL-1β-induced endothelial dysfunction. Neither tBHQ alone nor its combination with IL-1β did modify the contraction of K-KHS (results not shown).

In summary, these results indicate a beneficial role for Nrf2 activation on vascular alterations such as VSMC migration and proliferation and the endothelial dysfunction induced by IL-1β.

## Discussion

In the present study we found that TLR4 pathway contributes to the prooxidant and proinflammatory effects induced by IL-1β in VSMC, being these effects reduced by activation of the redox-sensitive transcription factor Nrf2. Thus, Nrf2 activation prevents the detrimental effects of this cytokine on VSMC migration, proliferation and vascular endothelial dysfunction, likely by interfering with the IL-1β-induced TLR4 pathway.

IL-1β is one of the most relevant proinflammatory cytokines belonging to the IL-1 family (Abbate et al., 2020). The binding of IL-1β to its receptor, mainly through MyD88, activates signalling pathways involving MAPKs as well as transcription factors such as NF-κB and AP-1 (Krishnan et al., 2014). IL-1β levels are increased in several pathologies that involve oxidative stress and inflammation, including some CVDs such as atherosclerosis and hypertension (Krishnan et al., 2014; Mai and Liao, 2020; Melton and Qiu, 2021). For instance, it has been described that IL-1β contributes to atherogenesis by initiation, formation, and growth of the atheroma plaques (Abbate et al., 2020). Accordingly, Bhaskar et al. (2011) have shown that blocking IL-1β-mediated activity reduces plaque formation and subsequent atherosclerosis progression. Additionally, IL-1β also participates in the pathogenesis of hypertension (Melton and Qiu, 2021). Thus, inhibition of IL-1β activity reduces blood pressure in models of DOCA/salt- (Ling et al., 2017) and angiotensin II-induced hypertension in mice (Akita et al., 2021). It is widely known that IL-1β increases ROS production, thus contributing to the progression of vascular damage related to those CVDs (Shen et al., 2016). In agreement with previous studies (Martín et al., 2012), we observed that IL-1β increased the NADPH oxidase activity and the subsequent production of O_2_^.^^−^ and H_2_O_2_ in VSMC. Furthermore, IL-1β contributes to the development of CVDs not only by inducing oxidative stress, but also due to its proinflammatory effects by promoting the expression of a variety of inflammatory mediators. Thus, IL-1β induces the activation of the vascular cell adhesion molecule 1 (VCAM-1), which recruits inflammatory cells to the VSMC at the atherosclerotic lesion (Wang et al., 1995) and promotes vascular calcification (Sánchez-Duffhues et al., 2019). In a previous report, we have shown that IL-1β also increases COX-2 expression in VSMC from SHR (Martín et al., 2012). COX-2 is the inducible isoform of COX enzyme that catalyses the formation of prostaglandins, whose expression and activation is induced by oxidative stress and inflammatory stimuli (Zhao et al., 2021). COX-2 acts as an inflammatory mediator by releasing vasoconstrictors which may induce cardiovascular dysfunction (Félétou et al., 2011; Bomfim et al., 2012; Zhao et al., 2021), playing a critical role in the endothelium-dependent contraction related to CVDs progression (Zhao et al., 2021). It has been described that AP-1, which can be activated by IL-1β (Shen et al., 2016), contributes to COX-2 expression in VSMC (Palacios-Ramírez et al., 2019). In our study, we found that IL-1β induces the nuclear translocation of the AP-1 subunit phospho-c-Jun as well as COX-2 protein expression in VSMC. Additionally, our results using the antioxidant apocynin and the hydrogen peroxide scavenger catalase confirm the role of oxidative stress in the IL-1β-induced proinflammatory effects.

TLR4 contribution to the vascular damage by activating inflammatory signalling pathways involving oxidative stress mechanisms, and its closely association with the pathogenesis of CVDs such as atherosclerosis and hypertension, have been described (De Batista et al., 2014; Yang et al., 2014; Hernanz et al., 2015; Qi et al., 2021). In addition, a possible connection between IL-1β and TLR4 has been suggested; thus, treatment with this cytokine activates the TLR4/NF-κB signalling *via* MyD88-dependent and -independent pathways (Liu et al., 2014; Gu et al., 2017), and this effect was abolished after TLR4 inhibition (Xu et al., 2020). We found that treatment of VSMC with IL-1β did not modify TLR4 expression, in agreement to that found by Chen et al. (2013). However, IL-1β increased the expression of its adapter protein MyD88. This effect of IL-1β on MyD88 protein expression was prevented by using the specific intracellular TLR4 inhibitor CLI-095, which disrupts the interactions of TLR4 with its adaptors (Matsunaga et al., 2011), suggesting that the TLR4/MyD88-dependent signalling pathway was activated by this cytokine in VSMC. As mentioned above, IL-1β increases COX-2 expression in VSMC. Activation of the TLR4 pathway mediates the transcription of COX-2, a well-known TLR4 target protein in VSMC (Bomfim et al., 2012; Biancardi et al., 2017). Accordingly, we found that the increase in COX-2 expression induced by IL-1β was reduced by CLI-095. In addition, the IL-1β-elicited increase of both O_2_^.^^−^ and H_2_O_2_ production was also reduced by the TLR4 inhibitor.

Under normal conditions, VSMC, the major component of the vascular wall, have a characteristic contractile phenotype; however, under oxidative stress conditions and excessive inflammation these cells turn to a dedifferentiated phenotype which makes them more prone to migrate and proliferate (Badran et al., 2020; Qi et al., 2021). Several studies have reported that inflammatory mediators such as IL-1β induces VSMC migration and proliferation (Martín et al., 2012; Wang et al., 2012; Eun et al., 2015), then promoting vascular damage development (Badran et al., 2020). Downregulation or inhibition of TLR4 has been shown to reduce angiotensin II-induced VSMC migration and proliferation (De Batista et al., 2014; Qi et al., 2021), and this would contribute to explain the beneficial effects of TLR4 blockade in vascular remodelling and mechanical alterations observed in hypertension (Hernanz et al., 2015). Here, we found that inhibition of TLR4 reduces IL-1β-induced VSMC migration and proliferation. Cytokines also induce cardiovascular damage by impairment of endothelial-dependent relaxations (Briones et al., 2005; Jimenez-Altayó et al., 2006), and its inhibition in many cases ameliorates vascular endothelial dysfunction, as described by Vallejo et al. (2014) for IL-1β. Here, we confirm that IL-1β impairs endothelial-dependent relaxation. More importantly, the general TLR4 inhibitor CLI-095 prevented the deleterious effect of IL-1β on endothelial function. The beneficial effect of inhibiting TLR4 on endothelium-dependent vasodilation has been described by our group and others in several models of hypertension (De Batista et al., 2014; Hernanz et al., 2015) and atherosclerosis (Chen et al., 2020). Although we have not analysed the mechanisms by which TLR4 blockade improves the IL-1β-induced endothelial dysfunction, the reduction of oxidative stress might contribute to this effect, as has been reported in the hypertension-associated endothelial dysfunction (Hernanz et al., 2015). All these results reveal an interaction between IL-1β and TLR4, although the exact mechanism for this needs further investigation.

As mentioned above, IL-1β increases ROS production. An excessive ROS production leads to a disruption of redox homeostasis and molecular damage (Xia et al., 2017; Griendling et al., 2021). To counteract high ROS levels, cells may activate antioxidant mechanisms, being the redox-sensitive transcription factor Nrf2 one of these mechanisms. Nrf2 protects against oxidative stress by inducing the expression of antioxidant proteins and phase II detoxification enzymes, including HO-1, NQO1 and SOD, in response to changes in the intracellular redox balance (Yamamoto et al., 2018). HO-1 overexpression in atherosclerotic lesions is considered to be protective (Kishimoto et al., 2019), whereas inhibition of this enzyme resulted in the progression of atherosclerosis (Ishikawa et al., 2012). Furthermore, Nrf2 activation may have a protective role in hypertension (Lopes et al., 2015; Guzik and Touyz, 2017; Xia et al., 2017; Wang et al., 2018). Therefore, activation of Nrf2 is a potential target against CVDs. In our study, we found that treatment of VSMC with IL-1β increased Nrf2 nuclear translocation and enhanced the expression of its downstream proteins, simultaneously reducing the expression of the Nrf2 repressor protein Keap1. This Nrf2 activation could be explained as a compensatory mechanism of the cell against the deleterious effect of this cytokine. Of note, the use of apocynin suggests that oxidative stress is involved in Nrf2 activation by IL-1β. Consistent with this, exogenous hydrogen peroxide also increased the nuclear translocation of Nrf2 as well as the protein expression of HO-1 and NQO1. It is known that oxidative modification of Keap1 cysteines results in a conformational change that leads to the detachment of Nrf2 from Keap1 and the inhibition of its ubiquitination; however, oxidative stress, by increasing the downstream Nrf2-driven gene p62/SQSTM1, activates specific autophagy of Keap1, and thus can modify its expression (Katsuragi et al., 2016).

tBHQ is a synthetic phenolic antioxidant widely used as selective Nrf2 activator. tBHQ acts modifying thiol groups of cysteines on the Keap1 protein, which induces a conformational change in Keap1; this leads to the release of Nrf2 from its repressor, therefore allowing induction of antioxidant and anti-inflammatory responses by this transcription factor (Zhang and Hannink, 2003). As expected, after VSMC incubation with tBHQ, increased nuclear Nrf2 translocation was found; in addition, tBHQ enhanced the protein expression of HO-1 and NQO1 while reduced Nrf2 inhibitor Keap1 expression. Pretreatment of IL-1β-stimulated VSMC with tBHQ induced similar effects on Nrf2 pathway to that induced by tBHQ alone; in addition, the effects of coincubation of VSMC with H_2_O_2_ and tBHQ on Nrf2 translocation and the expression of its downstream antioxidant enzymes HO-1 and NQO1 were also similar to incubation with tBHQ alone, indicating that the observed effects are those induced by the Nrf2 activator. On the other hand, we found reduced IL-1β-induced NADPH oxidase activity as well as IL-1β-mediated O_2_^.^^−^ and H_2_O_2_ production in cells pretreated with tBHQ, which denotes a protective role of Nrf2 activation against the prooxidant effects of IL-1β. It is known that H_2_O_2_ activates NADPH oxidase leading to further O_2_^.^^−^ production (Li et al., 2001; Hernanz et al., 2015). Herein we confirm these results and, consistent with the antioxidant effect of tBHQ, we observed that this activator also reduces the increased NADPH oxidase activity and the subsequent O_2_^.^^−^ production induced by the exogenous addition of H_2_O_2_ in VSMC.

In addition to the protective effect against oxidative stress, Nrf2 also has anti-inflammatory properties (Alonso-Piñeiro et al., 2021). Thus, activators of Nrf2 inhibit the transcription of proinflammatory cytokines and they are used as anti-inflammatory drugs (Muri et al., 2020). Therefore, the result of Nrf2 activation on the observed IL-1β-induced proinflammatory effects was analysed. Although tBHQ alone did not affect the expression of the proinflammatory proteins, it reduced the IL-1β-induced MyD88 expression as well as the increased phosphorylation of MAPKs (p-p38 and p-JNK1/2), which likely reduced p-c-Jun and COX-2 expression. This is consistent with a previous report showing that activation of HO-1 reduced AP-1/COX-2 expression (Subedi et al., 2019). Our results indicate that Nrf2 activation keeps against the detrimental proinflammatory effects of IL-1β. Previously we have shown that oxidative stress plays a role in the proinflammatory effects induced by IL-1β in VSMC; therefore, the antioxidant properties of tBHQ would contribute to explain the beneficial effect of Nrf2 activation on the proinflammatory pathways induced by the cytokine.

On the other hand, we found that Nrf2 activation by tBHQ reduces VSMC migration and proliferation induced by IL-1β, suggesting a protective role of this transcription factor on the cytokine-induced vascular remodelling. The fact that tBHQ also reduces the H_2_O_2_-induced cell migration allows us to propose that the antioxidant properties derived from Nrf2 activation would contribute to this effect. By contrast, H_2_O_2_ reduces VSMC proliferation, in agreement to that found by Nickenig et al. (2002), and this effect was further reduced by Nrf2 activation. Differences in the source of ROS, either endogenous or exogenous, as well as in the concentration might explain the different effects of ROS in cell proliferation.

Besides the previously mentioned action on the detrimental prooxidant and proinflammatory effects of IL-1β, tBHQ partially prevents the IL-1β-induced endothelial dysfunction. This is consistent with a previous study by Wang et al. (2018), which describes that tBHQ prevented angiotensin II-induced endothelial dysfunction by a Nrf2 dependent mechanism. Similarly, Lopes et al. (2015) found that sulforaphane corrected the impaired endothelial function in SHRSP.

Finally, we found that activation of Nrf2 with tBHQ reduces the effects of the TLR4 ligand LPS on MyD88 expression, NADPH oxidase activity and cell migration. These results, together to the previously mentioned reduction of IL-1β MyD88 expression by tBHQ, allow us to propose that protection of Nrf2 activation against the detrimental prooxidant and proinflammatory effects of IL-1β, are due, at least in part, to the interference with the TLR4 pathway activated by the cytokine, although further experiments are needed to confirm whether the signalling pathway involves MyD88-dependent or -independent mechanisms.

**FIGURE 8:**
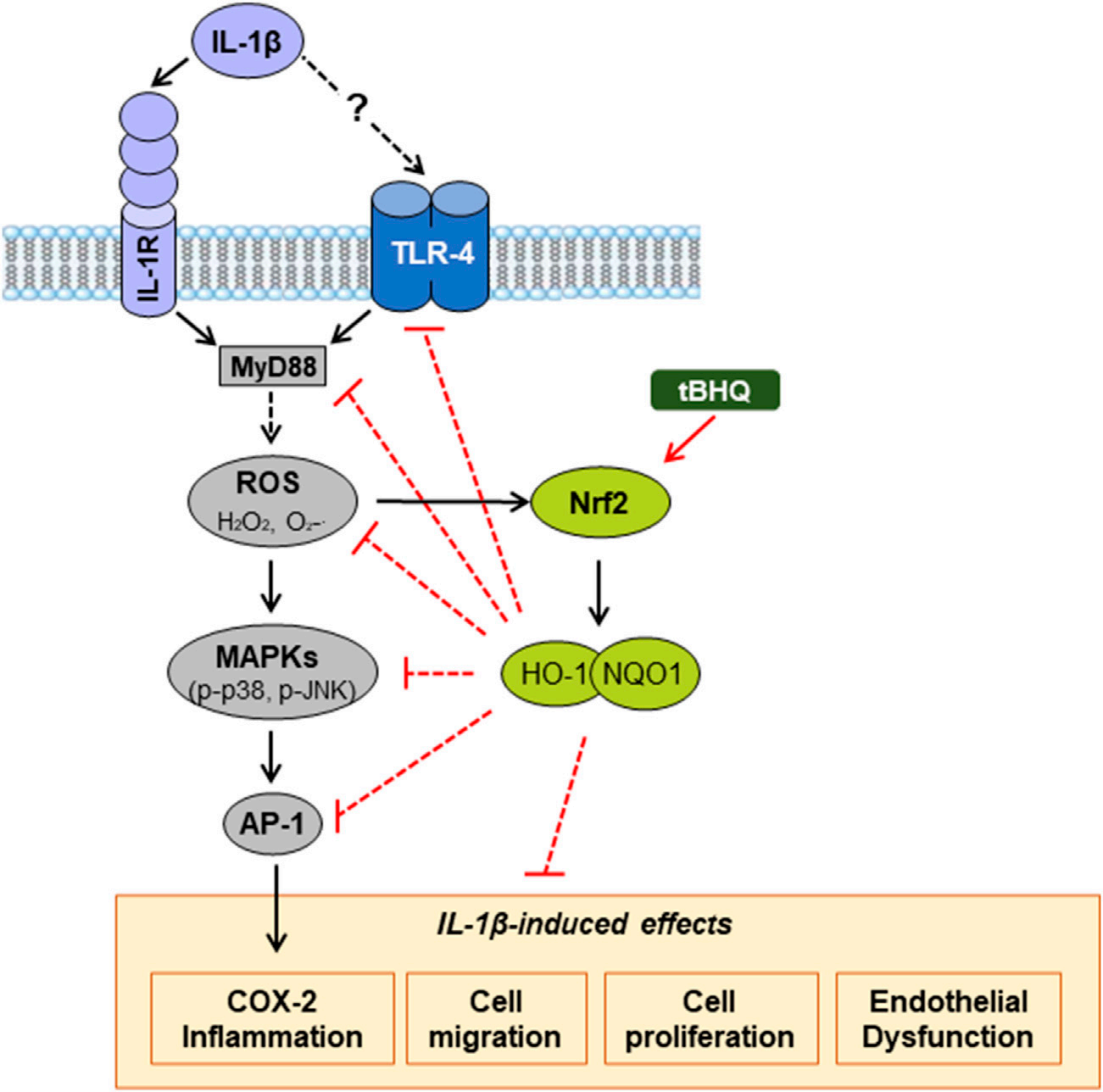
Proposed mechanism by which Nrf2 activation protects against vascular IL-1β effects. IL-1β increases oxidative stress and induces inflammation, cell migration and proliferation, as well as endothelial dysfunction, partially by a mechanism involving its interaction with TLR4 pathway. Nrf2 activation by tBHQ protects against these IL-1β vascular deleterious effects. The dashed lines indicate additional mechanisms unexplored in our study. IL-1β, Interleukin-1β; TLR4, toll-like receptor 4; MyD88, myeloid differentiation factor 88; ROS, reactive oxygen species; O_2_^.^^−^, superoxide anion; H_2_O_2_, hydrogen peroxide; tBHQ, tert-butylhydroquinone; Nrf2, nuclear factor-erythroid 2-related factor 2; HO-1, heme oxygenase-1; NQO1; NAD(P)H:quinone oxidoreductase 1; MAPK, mitogen-activated protein kinase; AP-1, activator protein-1; COX-2, cyclooxygenase-2.

In summary, the present study demonstrates that IL-1β elicits oxidative stress, inflammation, cell migration and proliferation, as well as endothelial dysfunction, by mechanisms involving its relationship with TLR4 pathway, although the exact mechanism by which IL-1β interacts with this receptor needs further investigation; however, we cannot rule out that some observed effects of IL-1β might also be produced by acting on its canonical IL-1R receptor (Figure 8). Moreover, our results reveal that activation of the redox-sensitive transcription factor Nrf2 induces vascular protection against IL-1β deleterious effects, at least in part, through interference with TLR4 pathway. Thus, by reducing TLR4 signalling pathway, Nrf2 activation reduces IL-1β-induced ROS generation and the increased expression of proinflammatory markers, in which oxidative stress participates. Furthermore, the activation of this transcription factor also prevents IL-1β-induced VSMC migration and proliferation, as well as the IL-1β-induced endothelial dysfunction (Figure 8). Therefore, our observations allow as to propose that activation of antioxidant and anti-inflammatory mechanisms such as Nrf2 might be an important therapeutic target in pathological conditions with oxidative and inflammatory components. However, the clinical application of Nrf2 activators for treatment of CVDs should be deeply analysed.

## Data Availability

The raw data supporting the conclusion of this article will be made available by the authors, without undue reservation.

## Glossary

### The following abbreviations are used in this document:

ACh: acetylcholine
AP-1: activator protein-1
COX-2: cyclooxygenase-2
CVDs: cardiovascular diseases
DCFH-DA: 2′,7′-dichlorofluorescein diacetate
DHE: dihydroethidium
DMSO: Dimethyl sulfoxide
DOCA: deoxycorticosterone acetate
FBS: fetal bovine serum
GAPDH: Glyceraldehyde-3-phosphate dehydrogenase
High Glucose-DMEM: High Glucose Dulbecco´s Modified Eagle Medium
**H**_**2**_ **O**_**2**_: hydrogen peroxide
HO-1: heme oxygenase-1
IL-1β: Interleukin-1β
JNK: c-Jun N-terminal kinase
Keap1: kelch-like ECH-associated protein 1
KHS: Krebs-Henseleit solution
LPS: lipopolysaccharide
MAPK: mitogen-activated protein kinase
MTT: 3-(4,5-dimethyl-2-thiazolyl)-2,5-diphenyltetrazolium bromide
MyD88: myeloid differentiation factor 88
NADPH oxidase: nicotinamide adenine dinucleotide phosphate (NADPH) oxidase
NQO1: NAD(P)H:quinone oxidoreductase 1
Nrf2: nuclear factor-erythroid 2-related factor 2
O_2_^.^^−^: superoxide anion
PBS: phosphate buffered saline
Phe: phenylephrine
ROS: reactive oxygen species
SHR: spontaneously hypertensive rats
SHRSP: stroke-prone spontaneously hypertensive rats
SOD: superoxide dismutase
tBHQ: tert-butylhydroquinone
TBP: TATA-binding protein
TLR4: Toll-like receptor 4
VSMC: vascular smooth muscle cells.
