# Supplementary material for: Regulation by Nrf2 of IL-1β-induced inflammatory and oxidative response in VSMC and its relationship with TLR4
Source: Front Pharmacol. 2023 Mar 2;14:1058488. doi: 10.3389/fphar.2023.1058488 (PMC10018188; doi:10.3389/fphar.2023.1058488)
Supplement: Supplementary file 1 [file Table1.docx]

Supplementary Material

## Supplementary Figures

**
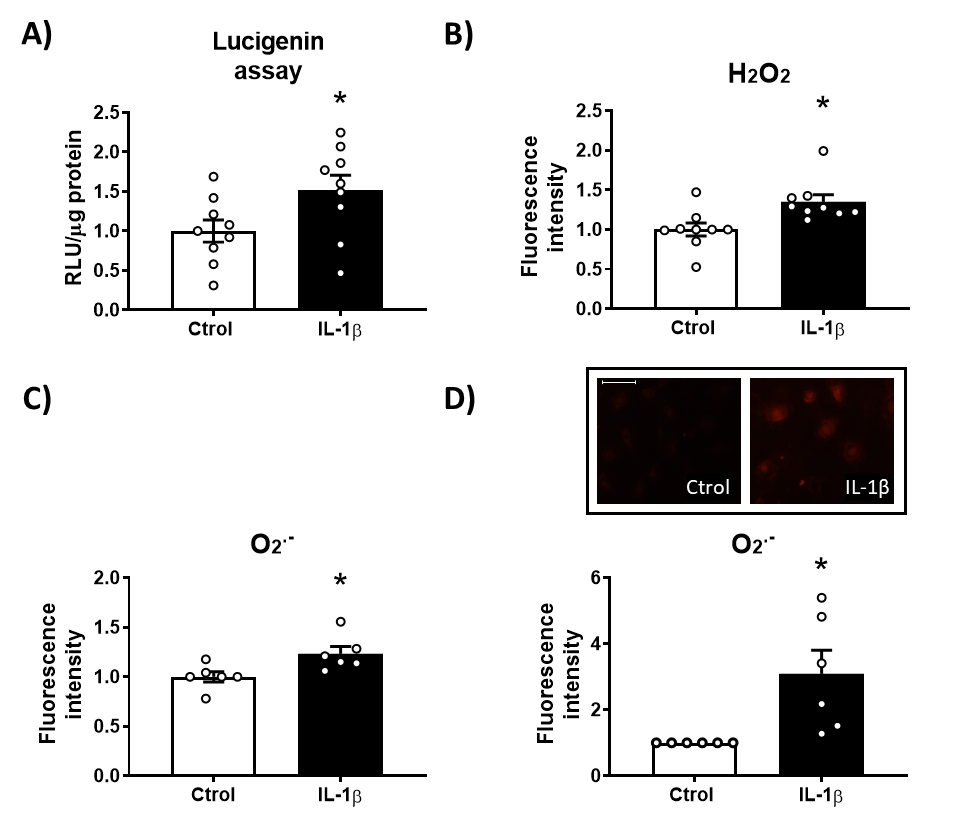
Supplementary Figure S1.** **Effects of IL-1β on oxidative stress.** Effect of Interleukin-1β (IL-1β, 10 ng/mL, 1 h) on NAPDH oxidase activity (**A**), hydrogen peroxide (H_2_O_2_) and superoxide anion (O_2_^.-^) production evaluated by flow cytometry (**B, C**), and O_2_^.-^ production evaluated by fluorescence microscopy (**D**) in vascular smooth muscle cells; representative fluorescent photomicrographs are also shown; images were captured with a fluorescence microscope; bar scale represents 100 μm. **p*< 0.05 vs. control by Student´s t-test (*n*= 6-10).


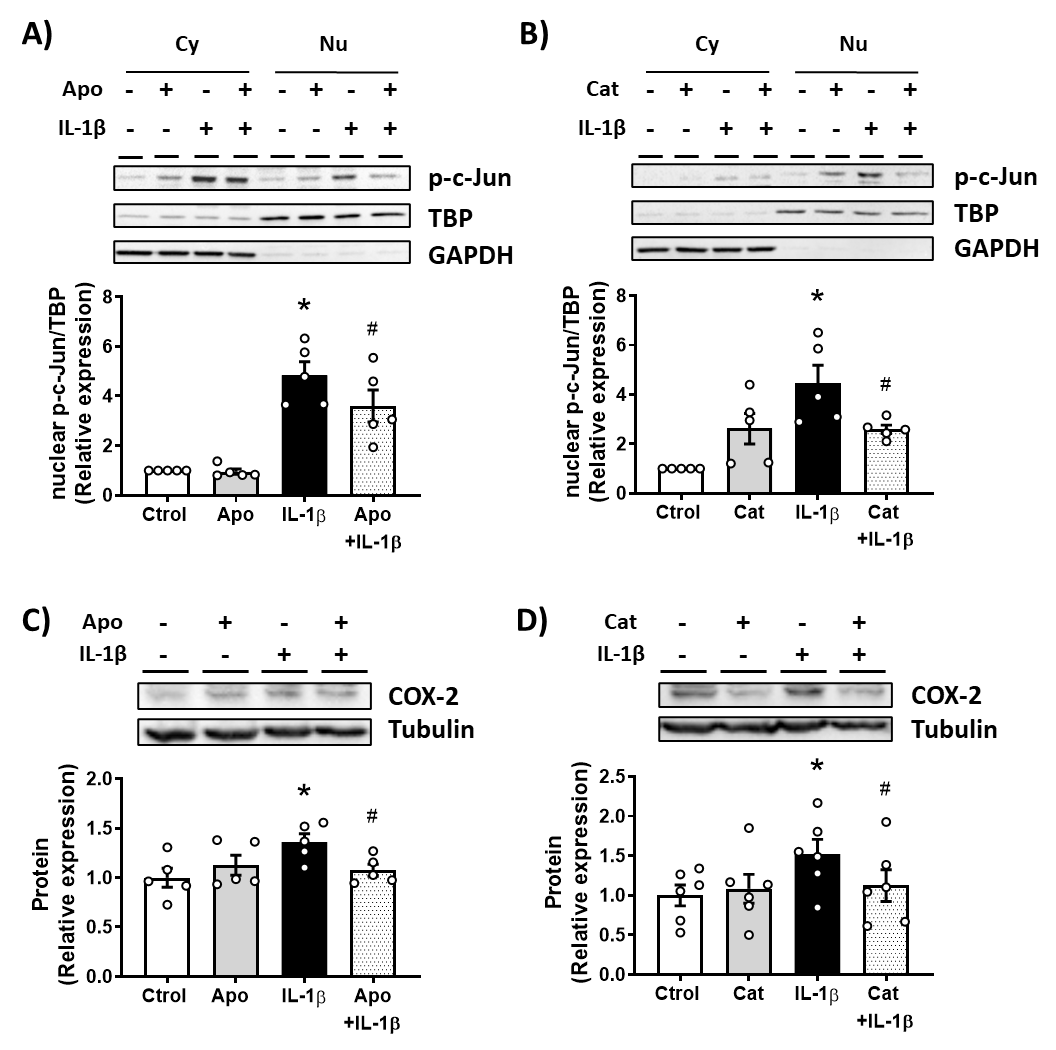


**Supplementary Figure S2. Role of oxidative stress in the IL-1β-induced effects on proinflammatory markers.** Effect of apocynin (Apo, 30 µM, 2 h) and catalase (Cat, 1000 U/mL, 2 h) on the Interleukin-1β (IL-1β, 10 ng/mL, 1 h)-induced nuclear p-c-Jun protein expression (**A, B**) in vascular smooth muscle cells; a representative blot of the cytosolic (Cy) and nuclear (Nu) expression is also shown; nuclear TATA-binding protein (TBP) and cytosolic GAPDH expressions are also shown to guarantee the successful cellular fractioning. Effect of Apo and Cat on cyclooxygenase (COX-2) protein expression induced by IL-1β (**C, D**). Representative blots are shown in upper panels. **p*< 0.05 vs. control; #*p*< 0.05 vs. IL-1β by Student´s t-test (*n*= 6-10).

**
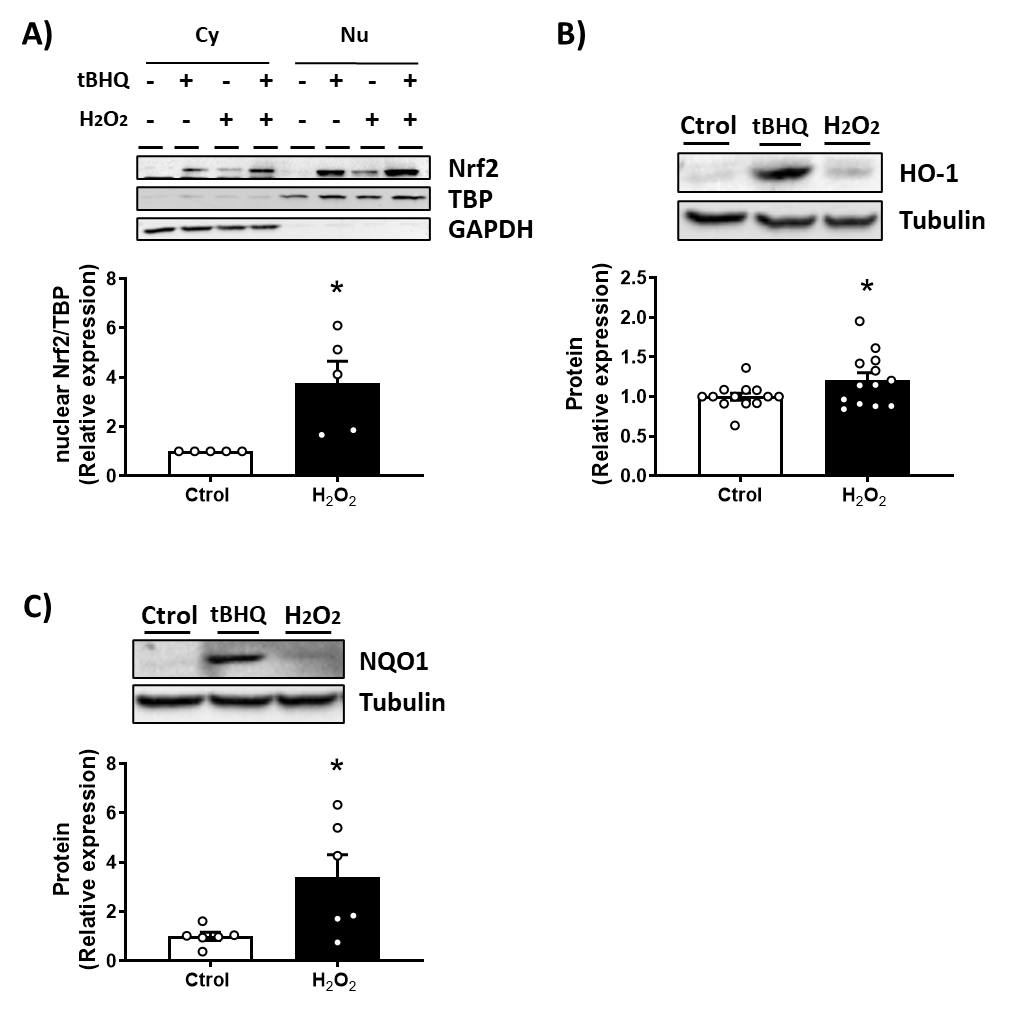
Supplementary Figure S3**. **Effects of H_2_O_2_ on the Nrf2 pathway.** Effect of hydrogen peroxide (H_2_O_2_,100 µM, 1h) on the nuclear Nrf2 (nuclear factor-erythroid 2-related factor 2) protein expression (**A**) in vascular smooth muscle cells; a representative blot of the cytosolic (Cy) and nuclear (Nu) expression is also shown; nuclear TATA-binding protein (TBP) and cytosolic GAPDH expressions are also shown to guarantee the successful cellular fractioning; the effect of tert-butylhydroquinone (tBHQ, 20 μM, 24 h) as positive control and tBHQ+H_2_O_2_ is also shown. Effect of H_2_O_2_ on heme oxygenase-1 (HO-1, **B**) and NAD(P)H:quinone oxidoreductase 1 (NQO1, **C**) protein expressions; tBHQ was used as positive control. Representative blots are shown in upper panels. *p< 0.05 vs. control by Student´s t-test (n= 5-10).

**
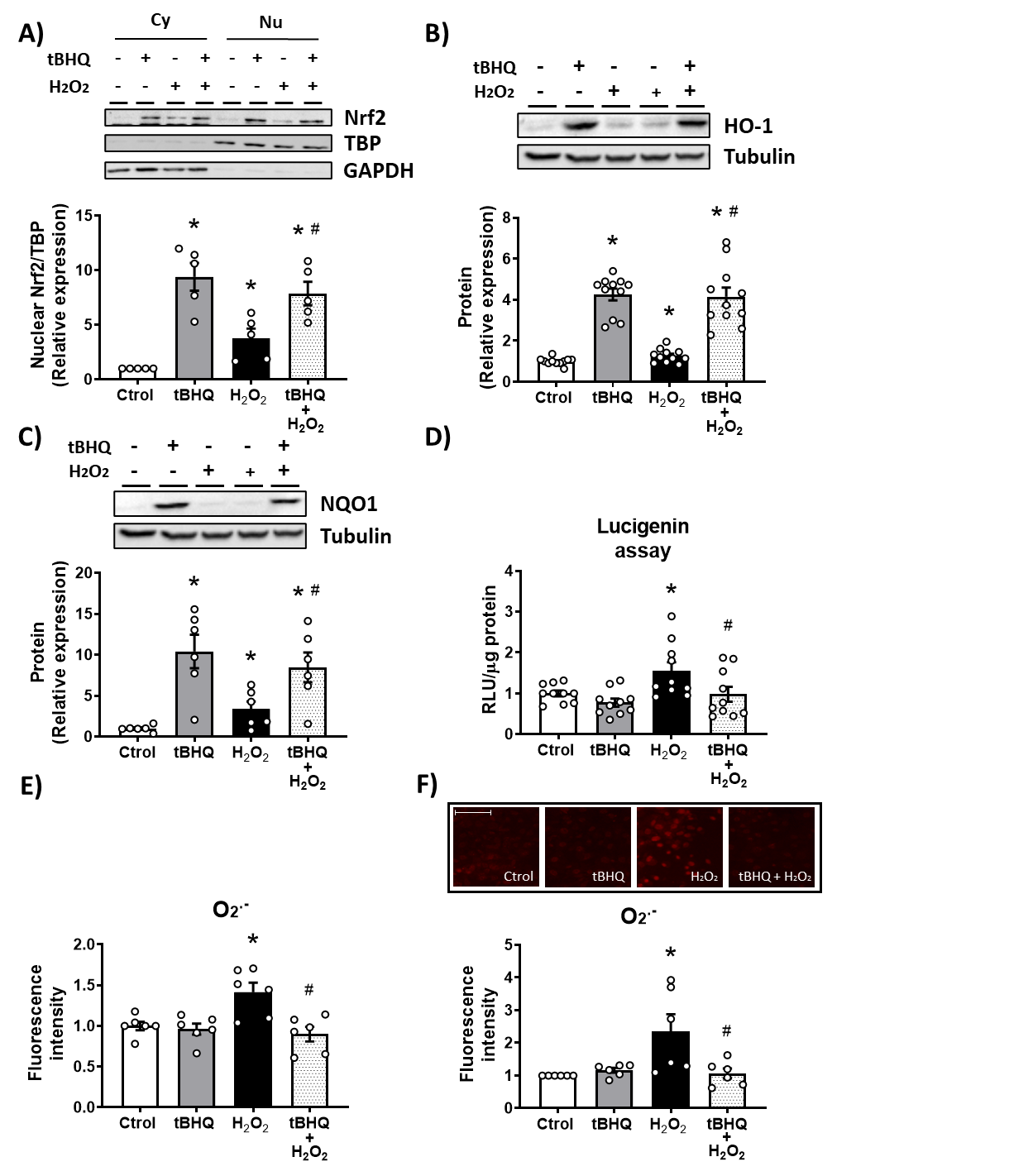
Supplementary Figure S4. Modulation by Nrf2 activation of the H_2_O_2_-induced effects on Nrf2 pathway and oxidative stress.** Effect of tert-butylhydroquinone (tBHQ, 20 µM, 24 h), hydrogen peroxide (H_2_O_2_, 100 µM, 1h) and tBHQ + H_2_O_2_ on the nuclear Nrf2 (nuclear factor-erythroid 2-related factor 2) protein expression (**A**) in vascular smooth muscle cells; a representative blot of the cytosolic (Cy) and nuclear (Nu) expression is also shown; nuclear TATA-binding protein (TBP) and cytosolic GAPDH expressions are also shown to guarantee the successful cellular fractioning. Effect of tBHQ, H_2_O_2_ and tBHQ + H_2_O_2_ on heme oxygenase-1 (HO-1, **B**) and NAD(P)H:quinone oxidoreductase 1 (NQO1, **C**) protein expression. Representative blots are shown in upper panels. Effect of tBHQ, H_2_O_2_ and tBHQ + H_2_O_2_ on NAPDH oxidase activity (**D**) and on superoxide anion (O_2_^.-^) production evaluated by flow cytometry (**E**) and fluorescence microscopy (**F**); representative fluorescent photomicrographs are also shown; images were captured with a fluorescence microscope; bar scale represents 100 μm. **p*< 0.05 vs. control; #*p*< 0.05 vs. H_2_O_2_ by Student´s t-test (*n*= 6-10).


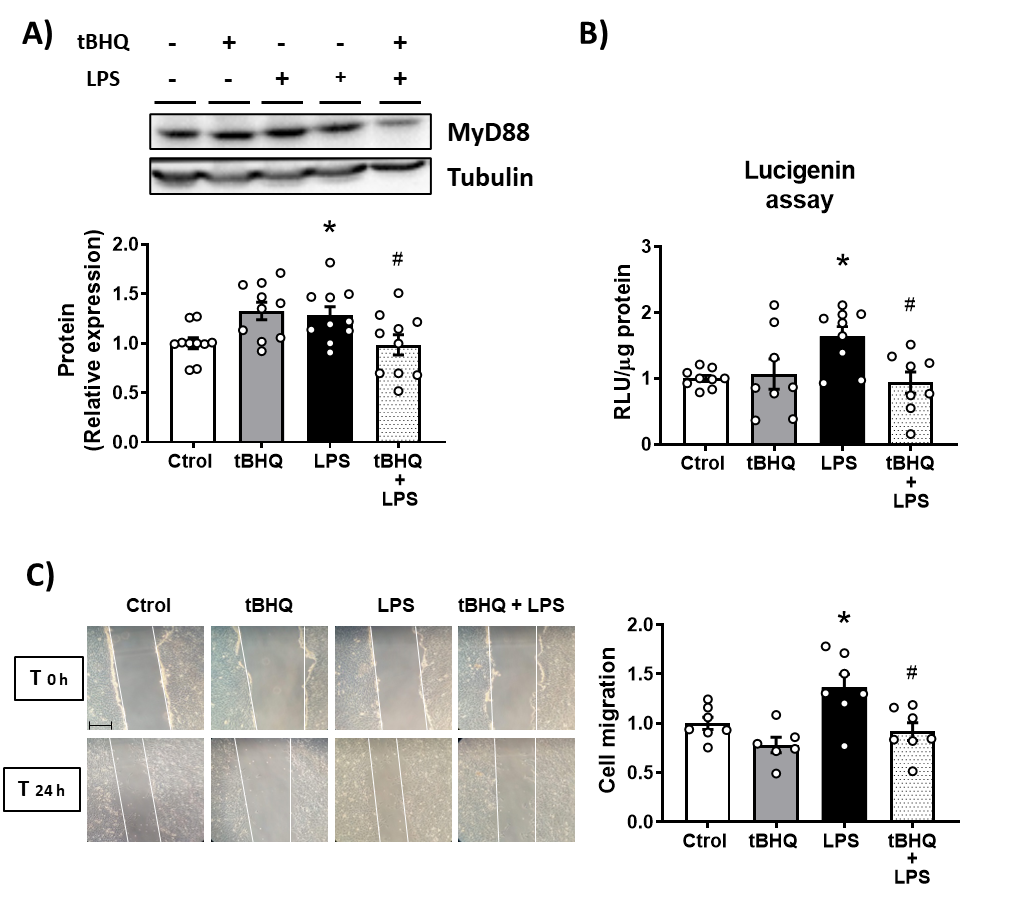


**Supplementary Figure S5. Modulation by Nrf2 activation of LPS-induced-effects.** Effect of tert-butylhydroquinone (tBHQ, 20 µM, 24 h) on lipopolysaccharide (LPS, 10 µg/mL, 3 h)-induced myeloid differentiation factor 88 (MyD88) protein expression (**A**), NAPDH oxidase activity (**B**) and cell migration (**C**) in vascular smooth muscle cells. Representative blot of protein expression is shown in upper panel. Images of cell migration by wound healing assay are included; bar scale represents 50 μm. **p*< 0.05 vs. control; #*p*< 0.05 vs. LPS by Student´s t-test (*n*= 5-12).

**
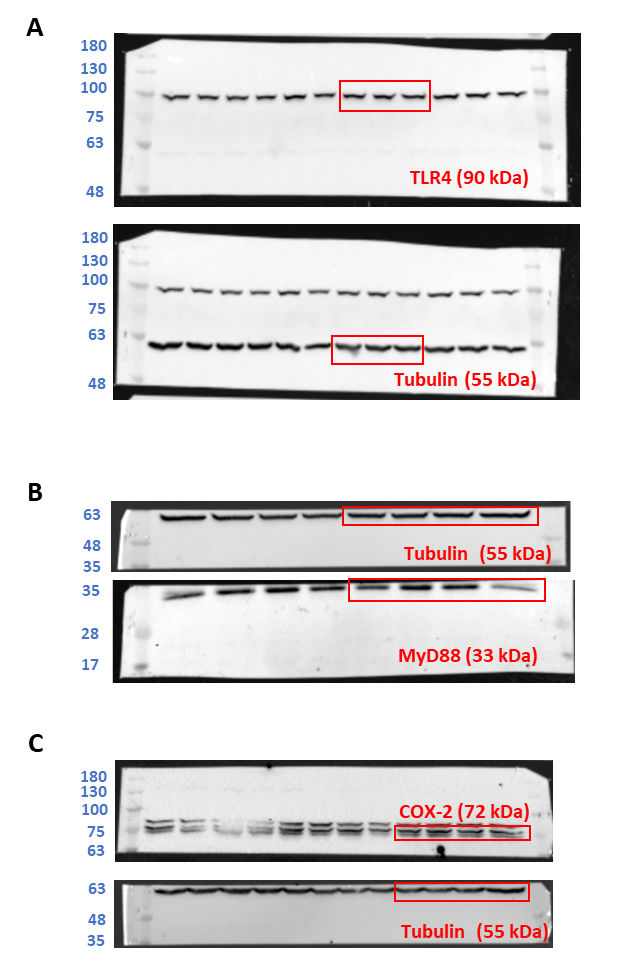
**

**Supplementary Figure S6.** Original uncropped images of blots shown in Figure 1. Bands in squares correspond to those shown in the corresponding Figure.

**
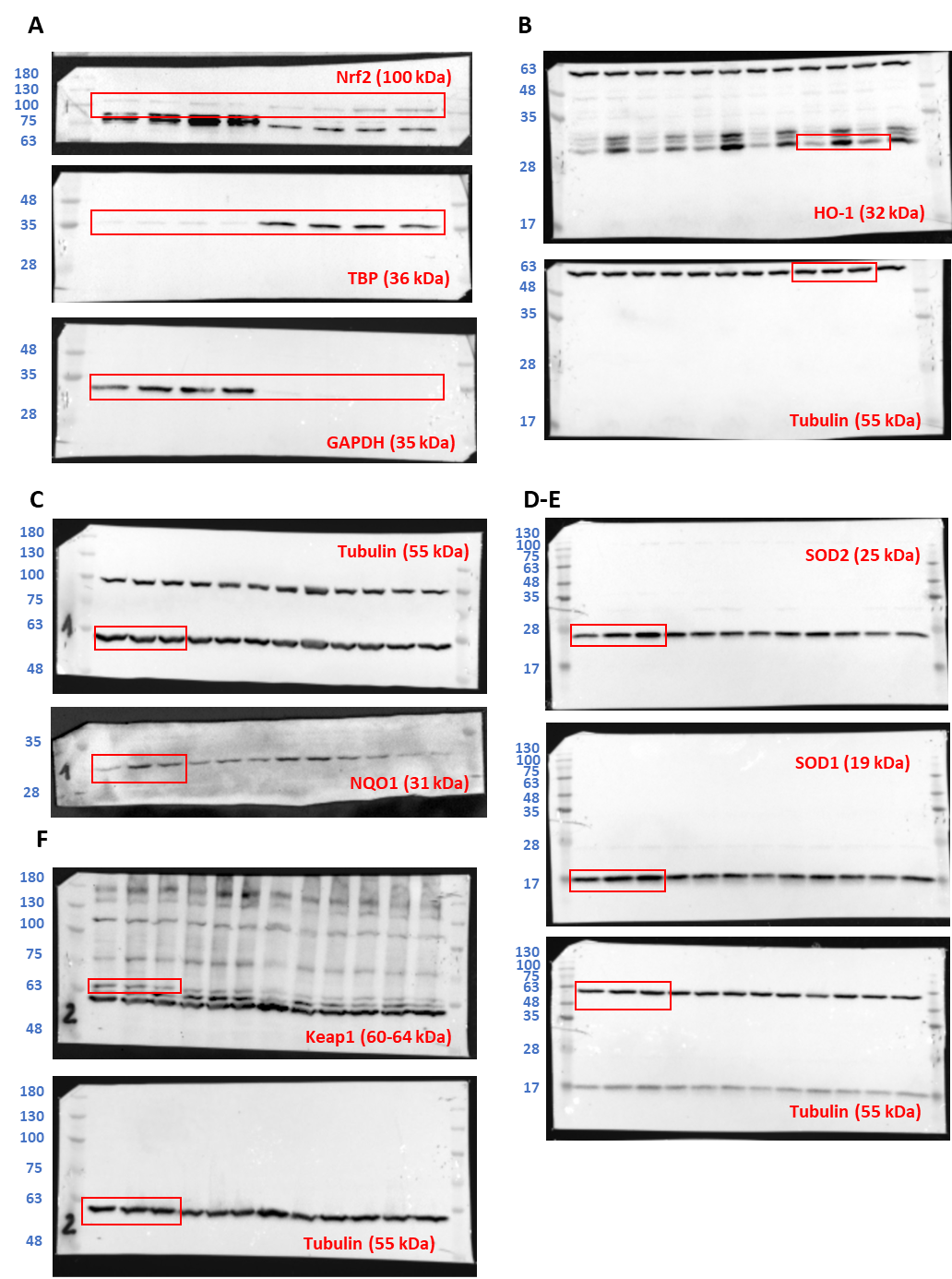
**

**Supplementary Figure S7.** Original uncropped images of blots shown in Figure 2. Bands in squares correspond to those shown in the corresponding Figure.

**
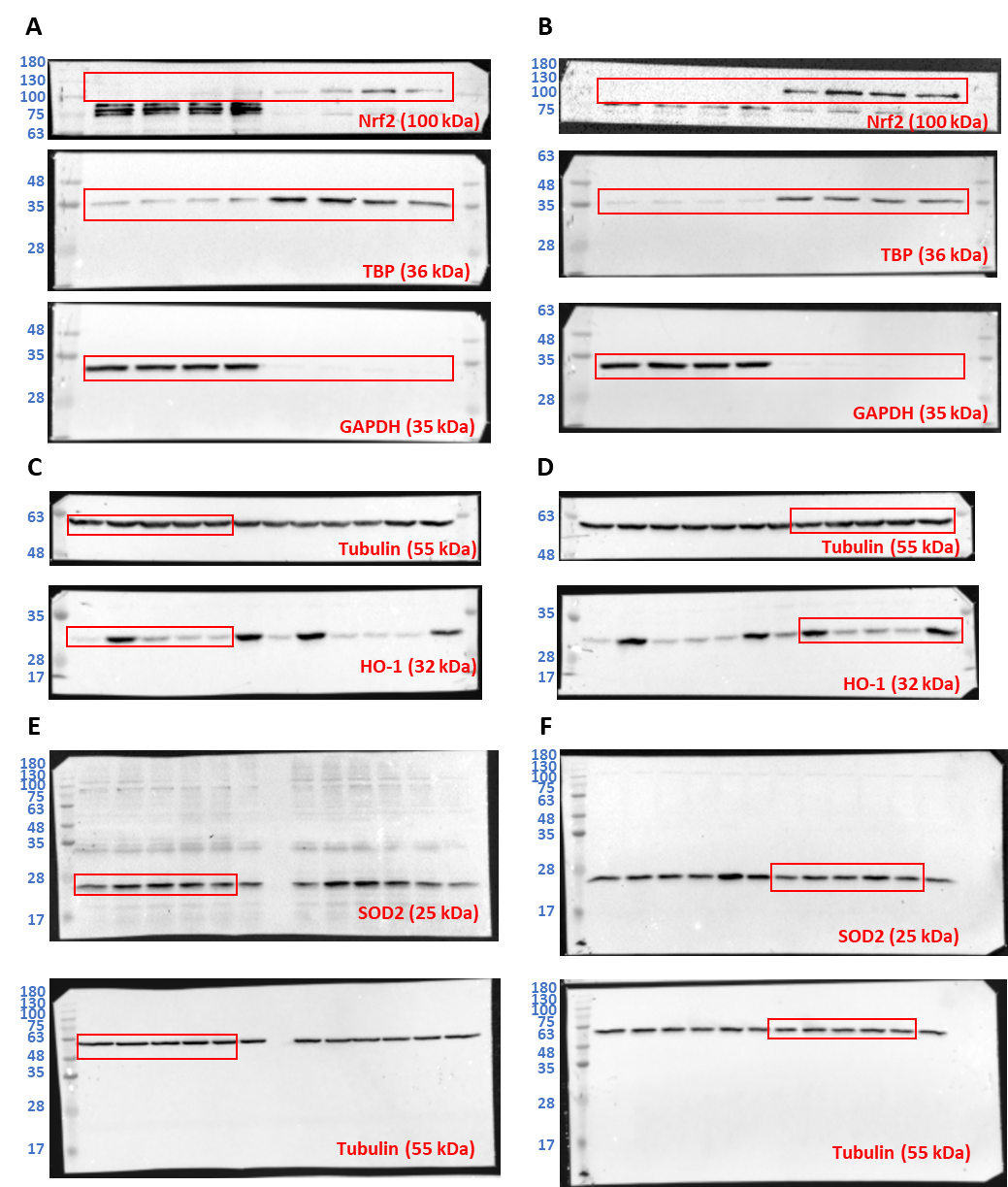
**

**Supplementary Figure S8.** Original uncropped images of blots shown in Figure 3. Bands in squares correspond to those shown in the corresponding Figure.

**
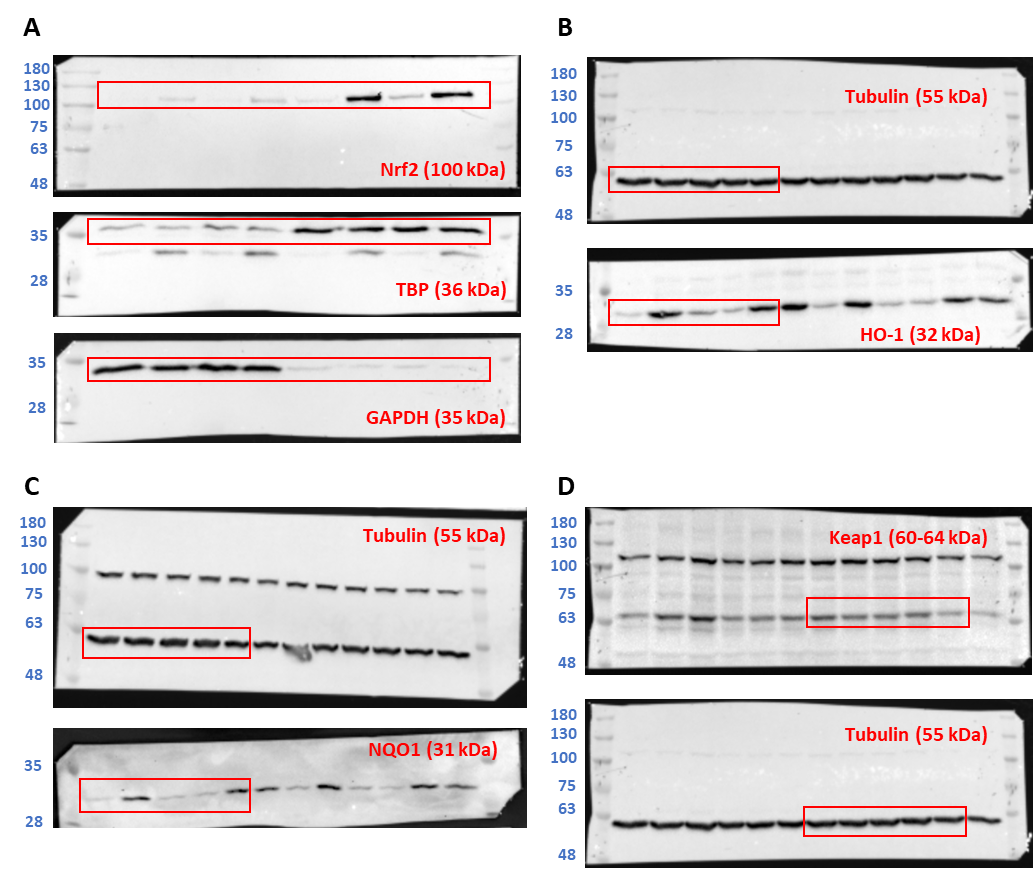
**

**Supplementary Figure S9.** Original uncropped images of blots shown in Figure 4. Bands in squares correspond to those shown in the corresponding Figure.

**
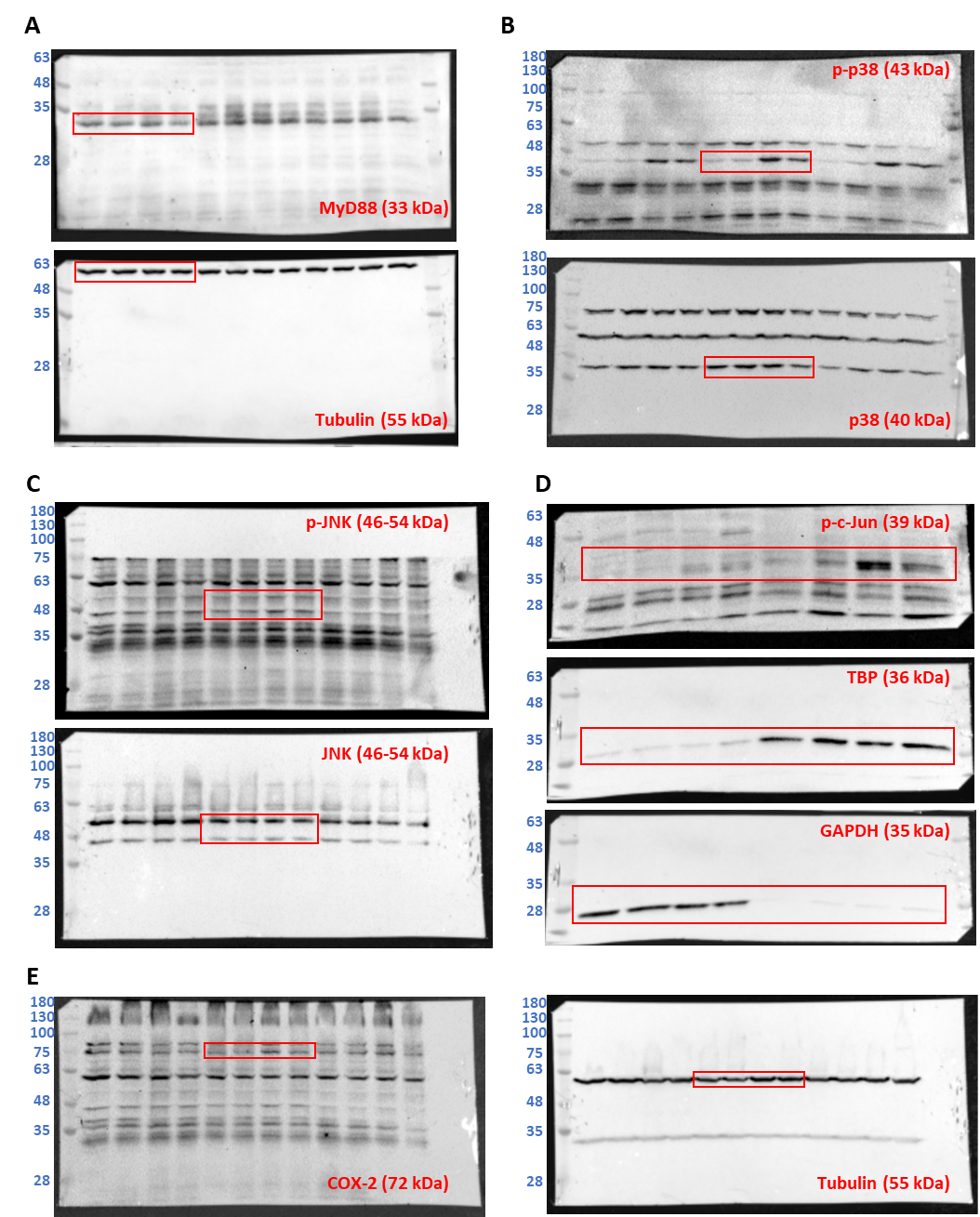
**

**Supplementary Figure S10.** Original uncropped images of blots shown in Figure 6. Bands in squares correspond to those shown in the corresponding Figure.


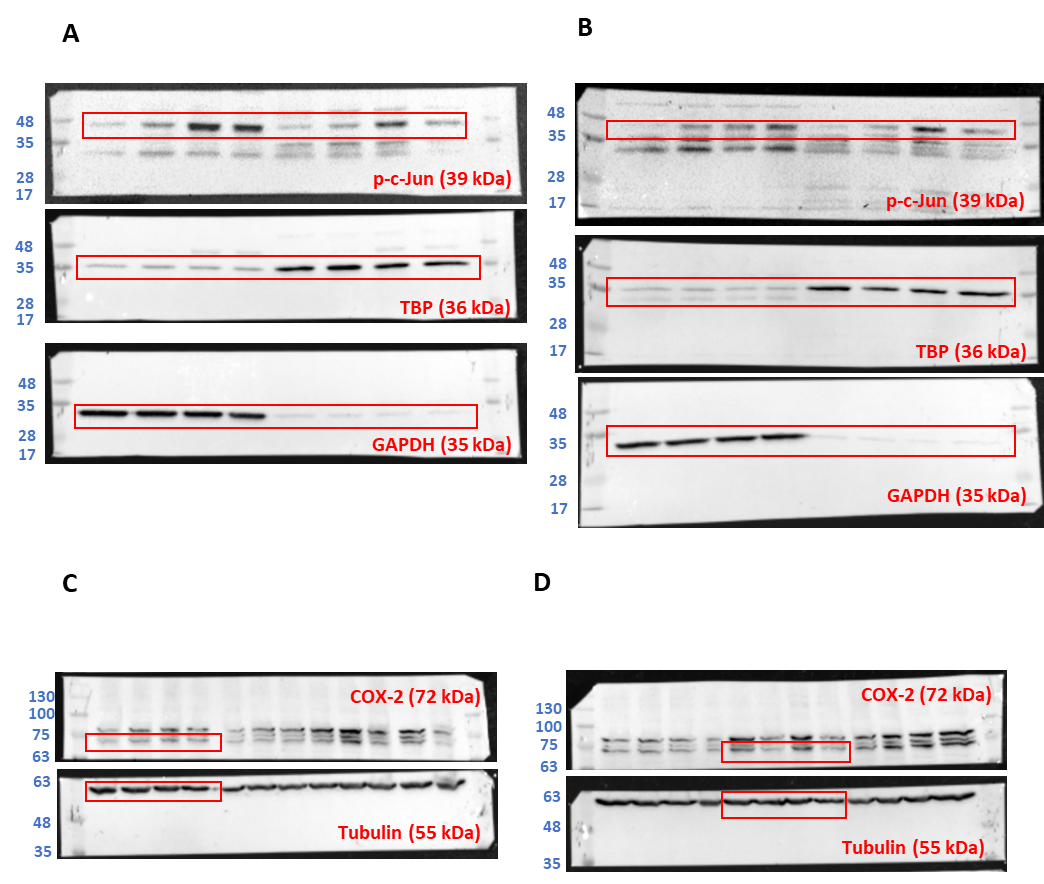


**Supplementary Figure S11.** Original uncropped images of blots shown in Supplementary Figure S2. Bands in squares correspond to those shown in the corresponding Figure.

**
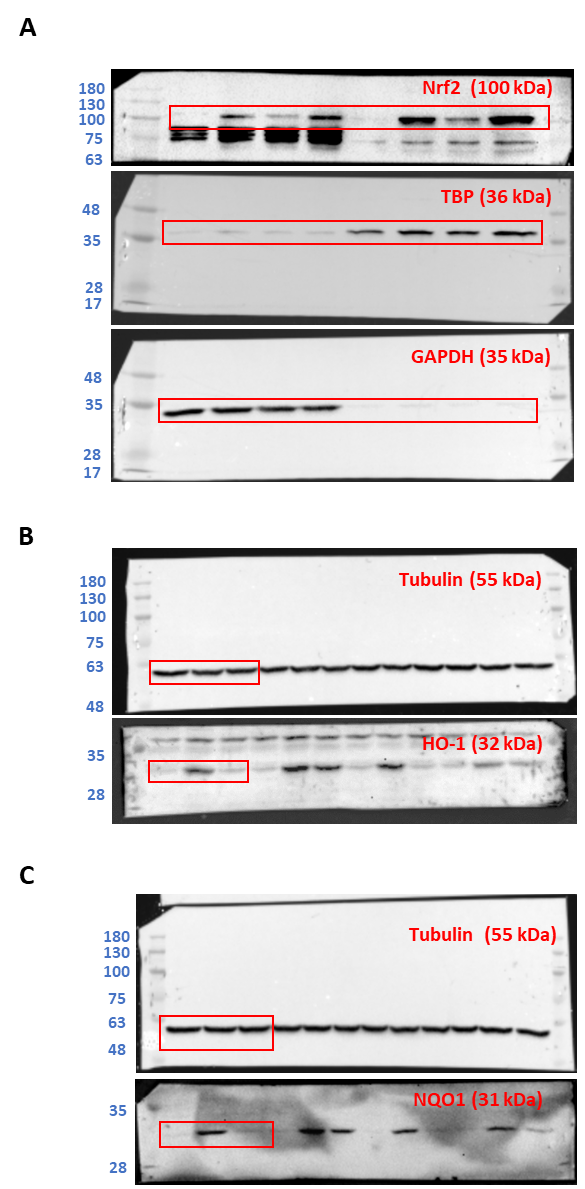
**

**Supplementary Figure S12.** Original uncropped images of blots shown in Supplementary Figure S3. Bands in squares correspond to those shown in the corresponding Figure.

**
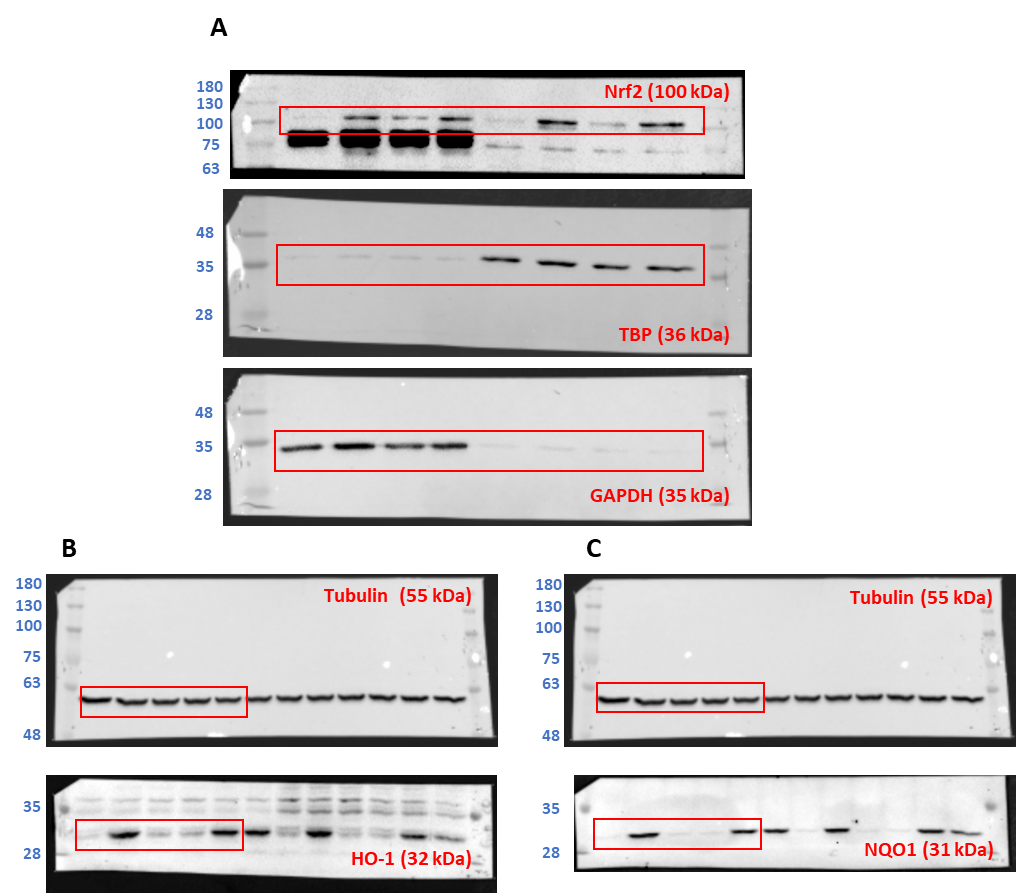
**

**Supplementary Figure S13.** Original uncropped images of blots shown in Supplementary Figure S4. Bands in squares correspond to those shown in the corresponding Figure.

**
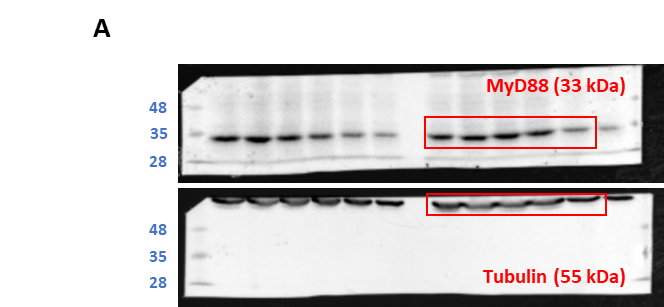
**

**Supplementary Figure S14.** Original uncropped images of blots shown in Supplementary Figure S5. Bands in squares correspond to those shown in the corresponding Figure.
